# Saprotrophic *Arachnopeziza* Species as New Resources to Study the Obligate Biotrophic Lifestyle of Powdery Mildew Fungi

**DOI:** 10.1111/1755-0998.70045

**Published:** 2025-10-03

**Authors:** Anne Loos, Ella Doykova, Jiangzhao Qian, Florian Kümmel, Heba Ibrahim, Levente Kiss, Ralph Panstruga, Stefan Kusch

**Affiliations:** ^1^ Unit of Plant Molecular Cell Biology, Institute for Biology I RWTH Aachen University Aachen Germany; ^2^ Institute of Bio‐ and Geosciences IBG‐2 Forschungszentrum Jülich Jülich Germany; ^3^ Centre for Crop Health, Institute for Life Sciences and the Environment University of Southern Queensland Toowoomba Queensland Australia; ^4^ Food and Wine Research Institute Eszterházy Károly Catholic University Eger Hungary; ^5^ Institute of Bio‐ and Geosciences ‐ Bioinformatics (IBG‐4) Forschungszentrum Jülich Jülich Germany

**Keywords:** evolution, fungi, genetic modification, genomics, lifestyle, powdery mildew

## Abstract

Obligate biotrophic plant pathogens like the powdery mildew fungi commit to a closely dependent relationship with their plant hosts and have lost the ability to grow and reproduce independently. Thus, at present, these organisms are not amenable to in vitro cultivation, which is a prerequisite for effective genetic modification and functional molecular studies. Saprotrophic fungi of the family *Arachnopezizaceae* are the closest known extant relatives of the powdery mildew fungi and may hold great potential for studying genetic components of their obligate biotrophic lifestyle. Here, we established telomere‐to‐telomere genome assemblies for two representatives of this family, *Arachnopeziza aurata* and *A. aurelia* . Both species harbour haploid genomes that are composed of 16 chromosomes at a genome size of 43.1 and 46.3 million base pairs, respectively, which, in contrast to most powdery mildew genomes that are transposon‐enriched, show a repeat content below 5% and signs of repeat‐induced point mutation. Both species could be grown in liquid culture and on standard solid media and were sensitive to common fungicides such as hygromycin and fenhexamid. We successfully expressed a red fluorescent protein and hygromycin resistance in *A. aurata* following polyethylene glycol‐mediated protoplast transformation, demonstrating that *Arachnopeziza* species are amenable to genetic alterations, which may be expanded to include gene replacement, gene modification, and gene complementation in the future. With this work, we established a potential model system that promises to sidestep the need for genetic modification of powdery mildew fungi by using *Arachnopeziza* species as a proxy to uncover the molecular functions of powdery mildew proteins.

**Abbreviations**
BLAST: Basic Local Alignment Search Tool
bp: base pair
BUSCO: Benchmarking Universal Single‐Copy Orthologs
CAZyme: carbohydrate‐active enzyme
CDS: coding sequence
ITS: internal transcribed spacer
KEGG: Kyoto Encyclopedia of Genes and Genomes
LINE: long interspersed nuclear element
LTR: long terminal repeat
MEA: malt extract agar
Mbp: million base pairs
nrDNA: nuclear ribosomal DNA
PCR: polymerase chain reaction
PDA: potato dextrose agar
PDB: potato dextrose broth
PEG: polyethylene glycol
PFAM: protein families
RFP: red fluorescent protein
RIP: repeat‐induced point mutation
rpm: revolutions per minute
TE: transposable element

## 1 Introduction

Powdery mildew is one of the most important and widespread plant diseases, visible as a greyish‐white tarnish or pustules on infected plant tissues such as leaves and stems (Glawe 2008). The disease is caused by the ascomycete powdery mildew fungi (family of *Erysiphaceae*), which comprise 19 genera and around 900 species infecting more than 10,000 angiosperm plant species (Braun and Cook 2012; Kiss et al. 2020; Kusch, Qian, et al. 2024).

The powdery mildews are obligate biotrophic plant pathogens, meaning they are fully dependent on living host plant tissue for growth and reproduction (Kemen et al. 2015; Spanu and Panstruga 2017). This lifestyle is associated with genomic hallmarks, including genome size expansion and gene losses in primary and secondary metabolism (Frantzeskakis et al. 2019; Spanu et al. 2010). The genome size of currently available near‐chromosome assemblies of powdery mildew fungi ranges from 76 to 212 million base pairs (Mbp), which is more than two times the average ascomycete genome size of around 37 Mbp (Kusch, Qian, et al. 2024; Mohanta and Bae 2015). The only known exception is *Parauncinula polyspora*, a powdery mildew fungus with a comparatively small (< 30 Mbp) genome that represents the most ancient extant lineage within the *Erysiphaceae* (Frantzeskakis et al. 2019; Vaghefi et al. 2022). In all other species with known genome assemblies, the size expansion can be attributed largely to transposable elements (TEs), which frequently cover 60%–80% of the genomes in powdery mildew fungi (Frantzeskakis et al. 2018; Kusch, Qian, et al. 2024; Müller et al. 2019; Spanu et al. 2010). Meanwhile, genes encoding components required for the assimilation of nitrogen and sulfur, anaerobic fermentation, and thiamine biosynthesis are missing in the powdery mildew genomes, signifying their dependence on the host to acquire important nutrients and metabolites (Frantzeskakis et al. 2019; Spanu et al. 2010; Sabelleck, Freh, et al. 2025). As a consequence, at present, it is not possible to routinely cultivate or reproducibly genetically modify powdery mildew fungi. Although alternative approaches such as host‐induced gene silencing (Nowara et al. 2010) and transient transformation of powdery mildew fungi (Martínez‐Cruz et al. 2018; Vela‐Corcía et al. 2015), mutagenesis approaches coupled with selection (Barsoum et al. 2020; Bernasconi et al. 2024), and experimental evolution (Kusch, Frantzeskakis, et al. 2024; Schwarzbach 1979) have been used successfully for studying aspects of powdery mildew virulence and evolution, the lack of cultivation and genetic modification protocols severely limits the functional molecular and genetic analysis of the obligate biotrophic lifestyle.

Based on multi‐gene and nuclear ribosomal DNA (nrDNA) phylogenetic analyses (Johnston et al. 2019; Vaghefi et al. 2022), the closest extant relatives of the *Erysiphaceae* known to date are members of the family *Arachnopezizaceae*. Five genera are currently assigned to the *Arachnopezizaceae*. The genera *Arachnopeziza*, *Arachnoscypha*, and *Eriopezia* have been included in the *Arachnopezizaceae* based on molecular phylogenetic analysis (Han et al. 2014), although *Arachnoscypha* was recently suggested to belong to the family *Hyaloscyphaceae* (Kosonen et al. 2020). Additionally, *Austropezia* and *Parachnopeziza* are assigned to the *Arachnopezizaceae* based on morphological features (Baral 2015; Han et al. 2014). The genus *Arachnopeziza* was first described in 1870 with the type species *A. aurelia* (Fuckel 1870), but *A. aurata* was later suggested to be the type species (Korf 1951). Currently, 12 (Kosonen et al. 2020) to 15 (Kirk et al. 2008) species are recognised within the genus *Arachnopeziza*, but several undescribed species are likely to exist.

The *Arachnopezizaceae* are saprobic fungi from the phylum Ascomycota and comprise species living on dead plant matter such as wood and litter (Ekanayaka 2019; Korf 1951). These fungi have thick‐walled hyaline hyphae. The asexual morph is hyphomycetous, that is, asexual spores (conidia) are formed from hyphae. During sexual reproduction, the fungi develop septate ellipsoid to fusoid ascospores (Ekanayaka 2019; Kirk et al. 2008; Korf 1951). The fruiting bodies are formed on a subiculum, which is an interconnected web‐like mycelium established by protruding hyphal elements and surrounding the apothecia (Kirk et al. 2008). The subiculum, together with septate spores, is a shared phenotypic characteristic of the *Arachnopezizaceae*, distinguishing them from other closely related families (Han et al. 2014; Korf 1951; Kosonen et al. 2020). Several *Arachnopeziza* species appear to be intimately associated with mosses such as *Sphagnum* and liverworts like *Ptilidium*, but the biological relevance of this relationship remains unclear (Kosonen et al. 2020; Stenroos et al. 2010).

Because the *Arachnopezizaceae* are phylogenetically the closest known extant relatives of the powdery mildew fungi (Johnston et al. 2019; Vaghefi et al. 2022) and exhibit a non‐pathogenic saprobic lifestyle (Korf 1951), we hypothesised that these fungi could constitute a suitable experimental system to establish molecular tools for genetic studies. We selected two species, *A. aurata* and *A. aurelia* , each represented by a strain deposited as a living culture at the CBS‐KNAW fungal culture collection (accession numbers CBS127674 and CBS127675, respectively), for analysis. We generated chromosome‐level genome assemblies and gene annotation resources, worked out cultivation conditions, and established a protoplast‐mediated transformation protocol, highlighting the potential of these fungal species to facilitate future molecular studies related to the obligate biotrophic lifestyle of the powdery mildew fungi.

## 2 Methods

### 2.1 Fungal Strains and Cultivation

The fungal strains *A. aurata* CBS 127674 and *A. aurelia* CBS 127675 were obtained in 2019 from the Westerdijk Fungal Biodiversity Institute at Utrecht University (CBS‐KNAW, https://wi.knaw.nl/). Both fungi were routinely cultivated on malt extract agar (MEA, 33.6 g L^−1^; Carl Roth GmbH, Karlsruhe, Germany) at 23°C in the dark. Agar plugs with fungal mycelium were transferred to fresh MEA plates with a drop of 200 μL of sterile H_2_O every 2–4 weeks to maintain the original strains. Liquid cultures were grown in potato dextrose broth (PDB, 26.5 g L^−1^; Carl Roth GmbH) at 28°C and 80 rpm (rpm) for 5–10 days. For testing different growth media, the fungi were further cultivated on potato dextrose agar (PDA, 39 g L^−1^; Sifin Diagnostics GmbH, Berlin, Germany), yeast peptone dextrose agar (YPDA, 65 g L^−1^; Carl Roth GmbH), or lysogeny broth agar (LB agar, containing 10 g L^−1^ tryptone, 5 g L^−1^ yeast extract, and 10 g L^−1^ sodium chloride; Carl Roth GmbH) at 23°C for 24 days. For testing different growth temperatures, the fungi were grown on MEA at 16°C, 23°C, 28°C, or 37°C for 24 days. Testing of long‐term storage at −80°C was done by adding glycerol at 10%, 20%, 25%, or 30% (v/v) final concentration to 1 mL of mycelium extracted from *A. aurata* and *A. aurelia* cultivated in PDB for 7 days and then flash‐freezing mycelia in liquid nitrogen. Mycelium was recovered by adding a droplet of thawed glycerol culture to MEA plates and cultivating fungi at 23°C in the dark. Wood and litter testing was done by collecting maple ( *Acer platanoides* ) twigs and leaves around the institute in Aachen (Germany; area around 50°46′38.9″N 6°02′46.9″E) and sterilising the twigs and leaves, as well as toothpicks, by steam sterilisation following a standard autoclaving procedure (121°C for 20 min). Toothpicks, twigs, or leaf segments were placed on MEA plates, and fungal mycelia were added adjacent to these; plates were incubated at 23°C in the dark for 3 months.

### 2.2 Antibiotic and Fungicide Susceptibility Assays

*A. aurata* and *A. aurelia* were cultivated at 23°C for 16 days on 1× MEA containing either of the following antibiotics or fungicides: hygromycin B (5–50 μg mL^−1^; Roche AG, Basel, Switzerland), fenhexamid (0.1–50 μg mL^−1^; Bayer AG, Leverkusen, Germany), geneticin G418 (5–50 μg mL^−1^; Santa Cruz Biotechnology, Dallas, USA), neomycin (5–50 μg mL^−1^; Duchefa Farma B. V., Haarlem, The Netherlands), kanamycin (5–400 μg mL^−1^; Carl Roth GmbH), streptomycin (5–400 μg mL^−1^; AppliChem GmbH, Darmstadt, Germany), ampicillin (5–400 μg mL^−1^; Duchefa Farma B. V.), and cefotaxime (5–400 μg mL^−1^; Carl Roth GmbH). Drug susceptibility was assessed by comparing mycelial growth at varying concentrations of the antibiotics or fungicides.

### 2.3 Protoplast Isolation

Fungal protoplasts were isolated based on a protocol for *Magnaporthe oryzae* (Leisen et al. 2020) with the following modifications: Mycelium balls were collected from liquid PDB cultures of *A. aurata* incubated at 80 rpm and 28°C after 7–14 days, and mycelia were shredded using a sterile blender. The shredded hyphae were further incubated in PDB at 80 rpm and 28°C for 3 days before transferring the mycelium to isoosmotic medium (1.2 M MgSO_4_, 10 mM NaPO_4_, pH 5.6 set with Na_2_HPO_4_) containing Glucanex (13 mg mL^−1^; Novozymes A/S, Bagsværd, Denmark) for 3 h at 28°C and 80 rpm. If protoplasts were detected via brightfield microscopy, they were filtered through sterile Miracloth (Merck KGaA, Darmstadt, Germany) and then washed using glacial TMS buffer (1 M sorbitol, 10 mM 3‐(N‐morpholino)propanesulfonic acid (MOPS), pH 6.3; Carl Roth GmbH). Next, protoplasts were centrifuged at 2000 × g and 4°C for 15 min and resuspended in 1 mL of TMSC buffer (TMS with 46 mM CaCl_2_) and placed on ice; protoplasts were then ready for PEG‐mediated transformation.

### 2.4 PEG‐Mediated Fungal Transformation

The plasmids pTK144 (hygromycin resistance) and pTelFen (fenhexamid resistance) were used as transformation vectors (Leisen et al. 2020; Wegner et al. 2022). The DNA fragments containing the transgenes (*mRFP*, *HPH*, and *FfERG27*, respectively) were amplified with the Phusion High‐Fidelity DNA Polymerase kit (New England Biolabs GmbH, Frankfurt a.M., Germany) using the oligonucleotides pTK144_FC_OE_Fw and pTK144_FC_OE_Rv (Table S1) and the following thermal profile: initial denaturation at 98°C for 30 s, 35 cycles of denaturation at 98°C for 10 s, annealing at 60°C for 30 s, and elongation at 72°C for 2.5 min, and a final extension cycle at 72°C for 5 min. Polymerase chain reaction (PCR) products were purified with the Monarch Genomic DNA Purification Kit (New England Biolabs GmbH).

Protoplasts in TMSC were placed on ice for 10 min. Approximately 6 μg of the amplified DNA was mixed with Tris‐CaCl_2_ (10 mM Tris, 1 mM EDTA, 40 mM CaCl_2_, pH 6.3) to a final volume of 30–60 μL. 120 μL of protoplasts was added to the DNA, which then rested on ice for 10 min before adding 180 μL PEG solution (0.6 g mL^−1^ PEG3350 (Sigma‐Aldrich, Munich, Germany), 1 M sorbitol, 10 mM MOPS, pH 6.3). Protoplasts were incubated at room temperature for 20 min. Then, protoplasts were regenerated in 50 mL of TB3 (0.2 g L^−1^ D(+)‐saccharose, 3 mg L^−1^ yeast extract; Carl Roth GmbH) at 28°C and 80 rpm for 3–5 days. Afterward, the fungal protoplasts were mixed with 50 mL of 2× MEA containing 10 μg mL^−1^ hygromycin B (Roche AG) or fenhexamid (Bayer AG) at 45°C and then poured into a petri dish. The plates were incubated at 23°C in the dark for 2–4 weeks. Colonies growing through the agar were transferred to new MEA plates containing hygromycin B or fenhexamid. Protoplasts without added DNA served as negative controls.

### 2.5 Diagnostic Polymerase Chain Reaction (PCR)

Diagnostic PCR was conducted using isolated genomic DNA with the OneTaq Quick‐Load DNA Polymerase system (New England Biolabs GmbH). PCRs were run with the following thermal profile: initial denaturation at 94°C for 30 s, 35 cycles of denaturation at 94°C for 30 s, annealing at 58°C–62°C for 15–60 s, and elongation at 68°C for 45 s, along with a final extension cycle at 68°C for 5 min. Oligonucleotides used in this work are listed in Table S1. PCR products were assessed via electrophoresis at 80–100 V in a 1% agarose gel in 1× TAE buffer (40 mM Tris–HCl, 20 mM acetic acid, 1 mM EDTA, pH 8.0) using ethidium bromide (Carl Roth GmbH) as an intercalating DNA dye. The 6× TriTrack loading dye (NEB) was used as a DNA loading dye. DNA bands were visualised on a GelDocTM XR+ (Bio‐Rad Laboratories GmbH, Feldkirchen, Germany).

### 2.6 Whole Transcriptome Shotgun Sequencing

*A. aurata* and *A. aurelia* were cultivated in PDB at 80 rpm and 28°C for 7 days. The mycelia balls were flash‐frozen in liquid nitrogen and crushed to a fine powder using a mortar and pestle, and RNA was isolated using the TRIzol extraction protocol following the manufacturer's instructions (Thermo Scientific, Karlsruhe, Germany). Genomic DNA removal was accomplished using RNase‐free DNase I (Thermo Scientific). We determined the quality of RNA samples using microcapillary electrophoresis (2100 BioAnalyzier system; Agilent, Santa Clara, CA, USA) and RNA quantity by spectrophotometry (NanoDrop; Thermo Scientific) and spectrofluorimetry (Qubit; Thermo Scientific), confirming high‐quality RNA for sequencing (RIN > 6.0, c[RNA] > 100 ng μL^−1^, m[RNA] > 1.5 μg). Stranded RNA sequencing with random oligomer priming, recovering total RNA, and depletion of plant/animal ribosomal RNA were performed by the service provider Novogene Europe (Cambridge Science Park, UK), yielding 150‐bp paired‐end reads. The raw reads (available at NCBI/ENA/DDBJ at project accession PRJNA1128938) were trimmed with Trimmomatic v0.39 (Bolger et al. 2014), and quality control of the reads was conducted using FastQC v0.12.1 (Babraham Bioinformatics, Cambridge, UK).

### 2.7 Whole Genome Sequencing and Genome Assembly

High molecular weight genomic DNA was obtained from *A. aurata* and *A. aurelia* , respectively, cultivated in PDB at 80 rpm and 28°C for 7–14 days. Mycelia balls were flash‐frozen in liquid nitrogen and crushed to a fine powder using a mortar and pestle. Then, DNA was isolated with a CTAB protocol according to Feehan et al. (2017) with the modifications indicated in Frantzeskakis et al. (2018). The DNA was further purified using the NucleoBond HMW DNA kit (Macherey‐Nagel, Düren, Germany); DNA integrity was tested via a 0.6% agarose gel using ethidium bromide as an intercalating dye and run at 30 V for 3 h. DNA quantity was determined using the Qubit dsDNA‐BR assay kit on a Qubit 4 (Thermo Fisher Scientific, Langerwehe, Germany).

DNA shotgun sequencing was performed using Illumina NovaSeq (NovaSeq 6000) technology with 1 μg input DNA at the service provider CeGaT (CeGaT, Tübingen, Germany), yielding 150‐bp paired‐end reads. We trimmed raw reads using Trimmomatic v0.39 (Bolger et al. 2014) and assessed read quality with FastQC v0.12.1 (Babraham Bioinformatics, Cambridge, UK). Long‐read sequencing was performed by MinION (Oxford Nanopore Technologies, Oxford, USA) with R9.4.1 flow cells and the Ligation Sequencing Kit SQK‐LSK112; basecalling was done using guppy v0.15.3. All raw reads are available at NCBI/ENA/DDBJ at project accession PRJNA1128938.

We generated draft genome assemblies using the long reads with Canu v2.2 (Koren et al. 2017), Flye v2.9.2 (Kolmogorov et al. 2019) with options ‘‐‐iterations 3 ‐‐threads 12 ‐‐genome‐size 42m ‐‐asm‐coverage 50 ‐m 10000’, and NextDenovo v2.5.2 (Hu et al. 2024) with configuration options ‘sort_options = ‐m 10g ‐t 8’ and ‘nextgraph_options = ‐a 1 ‐q 10 ‐E 5000’, and then merged the assemblies with quickmerge v0.3 (Chakraborty et al. 2016) to obtain the best draft assembly. We then remapped the Illumina short reads to the respective merged assemblies using the function ‘bwa mem’ of BWA v0.7.17‐r1188 (H. Li and Durbin 2009) and polished the assembly using pilon v1.24 (Walker et al. 2014).

We obtained basic assembly statistics using Quast v5.2.0 (Gurevich et al. 2013) and assembly quality estimations with CRAQ v1.0.9 (Li et al. 2023). Further, we identified the 5.8S, 18S, 28S nuclear ribosomal DNA (nrDNA) and ITS sequences using the nrDNA sequences of *A. aurata* CBS127674 and *A. aurelia* CBS127675 from GenBank (accessions MH864617.1, MH876055.1, MH864618.1, and MH876056.1; alignments at Files S1 and S2). Genome completeness was estimated using 1706 ascomycete core genes from the ascomycota_odb10 database with compleasm v0.2.6 (Huang and Li 2023; Simão et al. 2015).

### 2.8 Telomere Identification

Telomeres were manually identified at the ends of assembled contigs as telomeric repeats 5′‐TTAGGG‐3′ or 3′‐CCCTAA‐5′. To complete the sequence ends where telomeric repeats were not found, we used teloclip v0.0.4 (https://github.com/Adamtaranto/teloclip). Briefly, we mapped the long MinION nanopore reads to the respective assembly using Minimap2 v2.26‐r1175 (H. Li 2018) to retrieve nanopore reads at both ends of the assembly sequences containing telomeric repeats (5′‐TTAGGG‐3′) with options ‘‐k 20 ‐ax map‐ont’ and parsed the SAM files with SAMtools v1.18 (H. Li et al. 2009). Then, teloclip with options ‘‐‐motifs TTAGGG, TTAAGGG ‐‐matchAny’ was used to filter reads mapping to chromosome ends, and with options ‘‐‐extractReads ‐‐extractDir SplitOverhang’ to extract these reads. Then, the reads of each chromosome end were aligned via multiple sequence alignment using MAFFT v7.520 (Katoh and Standley 2013), and Jalview v2.11.3.2 (Waterhouse et al. 2009) was employed to manually identify and extend scaffold ends via read alignment until the last aligning telomeric repeat, where available.

### 2.9 Analysis of Genome Synteny

We used the functions nucmer for genome alignment and dnadiff, delta‐filter with option ‘‐l 1000’, and show‐coords with ‘‐c ‐l ‐L 1000’ for filtering from the MUMmer v4.4.0 package (Kurtz et al. 2004). We employed RIdeogram v0.2.2 (Hao et al. 2020) in R v4.3.1 (R Core Team 2018) (www.r‐project.org/) to visualise synteny, inversions, and translocations between the genome assemblies of *A. aurata* and *A. aurelia* .

### 2.10 Genome Map Visualisation

We visualised the genomic maps of *A. aurata* and *A. aurelia* as a circos diagram annotated with telomeres, centromeres, GC content, TE, and gene density. First, we used BEDtools v2.31.0 (Quinlan and Hall 2010) to generate 5,000‐bp sliding windows with the function ‘makewindows’. Then, we calculated the GC content in each window with ‘bedtools nuc’ and further counted the number of annotated TEs and genes in each window via ‘bedtools intersect’. We searched for potential candidate centromeric regions by identifying windows covering between 30,000 and 150,000 bp where the gene content was zero or near zero and the TE content was at least 5 TEs per window. We then used circlize v0.4.10 (Gu et al. 2014) in R v4.3.1 (R Core Team 2018) (www.r‐project.org/) to visualise the genomic maps (see 03.circos_plot.md at https://github.com/stefankusch/arachnopeziza_analysis for a detailed script).

### 2.11 Annotation of Mitochondrial Genomes

We used the online server of MFannot at https://megasun.bch.umontreal.ca/apps/mfannot/ accessed in 07/2024 (Lang et al. 2023) with the genetic code ‘4 Mold, Protozoan, and Coelenterate Mitochondria; Mycoplasma/Spiroplasma’ to discover and annotate mitochondrial genetic components. In addition, we used BLASTN and TBLASTN via NCBI BLAST+ v2.11.0 (Altschul et al. 1997) to search for coding genes and noncoding RNAs typically found in fungal mitochondrial genomes, that is, ATP synthase subunit 6 (*atp6*, GenBank accession AGN49024.1), *atp8* (RKF65626.1), *atp9* (AGN49018.1), cytochrome c oxidase assembly factor 3 (*coa3‐cc*, KAK6608516.1), mitochondrial cytochrome b (*cob*, ACL50595.1), cytochrome c oxidase subunit 1 (*cox1*, QPZ56210.1), cox2 (AGN49030.1), cox3 (AGN48999.1), NADH dehydrogenase subunit 1 (*nad1*, AGN49020.1), *nad2* (AGN49027.1), *nad3* (AGN49029.1), *nad4* (AGN49012.1), *nad4L* (AGN48994.1), *nad5* (AGN48996.1), *nad6* (AGN48998.1), mitochondrial small ribosomal subunit (rns), mitochondrial large ribosomal subunit (rnl), RNA component of ribonuclease P (*rnpB*, X93307.1), and ribosomal protein S3 (*rps3*, AGN49003.1).

### 2.12 Annotation of Transposable Elements

We used the TEtrimmer pipeline (Qian et al. 2025) with the EDTA2‐generated repeat libraries (Ou et al. 2019) as input to generate a consensus TE library for genome‐wide annotation of TEs in the assemblies of *A. aurata* and *A. aurelia* . The two resulting libraries were the user‐defined libraries for genome‐wide TE annotation with RepeatMasker v4.0.9 (http://www.repeatmasker.org) (Smit et al. 2016), yielding TE occupancy statistics, a TE annotation file, and the soft‐masked genome assemblies. The sequence divergence of TE classes was calculated as described previously (Frantzeskakis et al. 2018, 2019).

### 2.13 RIP Analysis

Dinucleotide frequencies were calculated using RIPCAL v2.0 (Hane and Oliver 2008), using FASTA sequences of the whole genome assembly (without the mitochondrial genome), coding sequences determined with BRAKER3, and repeat elements annotated with RepeatMasker (see above), that is, *Ty1*/*Copia*, *Ty3*/*mdg4*, and DNA transposons as input. Then, the dinucleotide frequency indices were calculated in R v4.3.1 (R Core Team 2018) (www.r‐project.org/) (see 03.ripcal.md at https://github.com/stefankusch/arachnopeziza_analysis for a detailed script).

### 2.14 Gene Annotation

We used BRAKER3 v3.0.8 (Bruna et al. 2023; Gabriel et al. 2021, 2023) for evidence‐based gene annotation of both *A. aurata* and *A. aurelia* . The respective RNA‐seq data obtained in this work and the OrthoDB v11 Fungi protein dataset (https://www.orthodb.org/) (Kuznetsov et al. 2023) served as evidence datasets for BRAKER3 predictions. Reads were prepared for annotation by mapping to the respective genome assembly with HISAT2 (Kim et al. 2015) with ‘‐‐max‐intronlen 1000 ‐k 10’ and parsing the SAM files with SAMtools v1.18 (H. Li et al. 2009). We assessed the completeness of the gene annotations using BUSCO v5.5.0 (Simão et al. 2015) with ‘‐m protein’ and the ascomycota_odb10 database.

We performed functional gene annotations using hmmscan from HMMer v3.4 (Potter et al. 2018) and InterProScan v5.73‐104.0 (Jones et al. 2014) to predict functional protein domains, TMHMM v2.0c (Krogh et al. 2001) to detect putative transmembrane domains, SignalP5.0 (Almagro Armenteros et al. 2019) for secretion signals, EffectorP3.0 (Sperschneider and Dodds 2021) for prediction of apoplastic/cytoplasmic effectors, dbCAN3 search at https://bcb.unl.edu/dbCAN2/ (accessed 07/2024) (Zheng et al. 2023) for the identification of candidate carbohydrate‐active enzymes (CAZymes), antiSMASH v7.1.0 (Blin et al. 2023; Medema et al. 2011) to find genes encoding enzymes for secondary metabolic compounds, and primary metabolism components were annotated using GhostKOALA to identify components and KEGG Mapper to reconstruct pathways via https://www.kegg.jp/ (accessed 04/2025) (Kanehisa et al. 2025; Kanehisa and Goto 2000). Orthologous proteins were identified using OrthoFinder v2.5.5 (Emms and Kelly 2019).

Secreted ribonuclease‐like proteins in the proteomes of *A. aurata* and *A. aurelia* were analysed as follows: We extracted the sequences of putative secreted proteins containing PFAM domains (PF00445, PF06479) or InterPro domains (IPR036430, IPR016191) from the Ribonuclease T2‐like superfamily. We used these sequences, that is, AAURAT_002659‐RB, AAURAT_005358‐RA, AAURAT_010638‐RA, and AAURAT_011677‐RA for *A. aurata* and AAUREL_003350‐RA, AAUREL_011122‐RA, AAUREL_004672‐RA for *A. aurelia* , as queries for a protein BLAST (BLASTP) against the non‐redundant protein database (nr on https://blast.ncbi.nlm.nih.gov/Blast.cgi accessed 04/2025) subset for a search within the Heliotales (taxonomy ID 5178) and randomly extracted protein sequences with high similarity to each of the queries. In addition, we included the sequences of three canonical RNase‐like effector proteins, which were PM2 of *B. graminis* f.sp. *tritici* and CSEP0064 and CSEP0264 of *B. hordei* (Spanu 2017). We conducted multiple sequence alignment using the MUSCLE algorithm and phylogenetic reconstruction using the function ‘build’ of ETE3 3.1.3 (Huerta‐Cepas et al. 2016) on the GenomeNet website (https://www.genome.jp/tools/ete/; accessed 04/2025). The maximum likelihood (ML) tree was inferred using RAxML v8.2.11 with model PROTGAMMAJTT and default parameters (Stamatakis 2014).

### 2.15 Data Analysis and Visualisation

The R v4.3.1 software environment (R Core Team 2018) (www.r‐project.org/) was used for data analysis and plotting. Data analysis was facilitated by the packages tidyverse v2.0.0, dplyr v1.1.2, reshape2 v1.4.4, and scales v1.2.1. Bar plots and histograms were created using the R package ggplot2 v3.4.2 (Wickham 2009). Synteny plots were generated with RIdeogram v0.2.2 (Hao et al. 2020) and circos plots with circlize v0.4.10 (Gu et al. 2014).

## 3 Results

### 3.1 *Arachnopeziza* Species Grow Under In Vitro Culture Conditions

**FIGURE 1:**
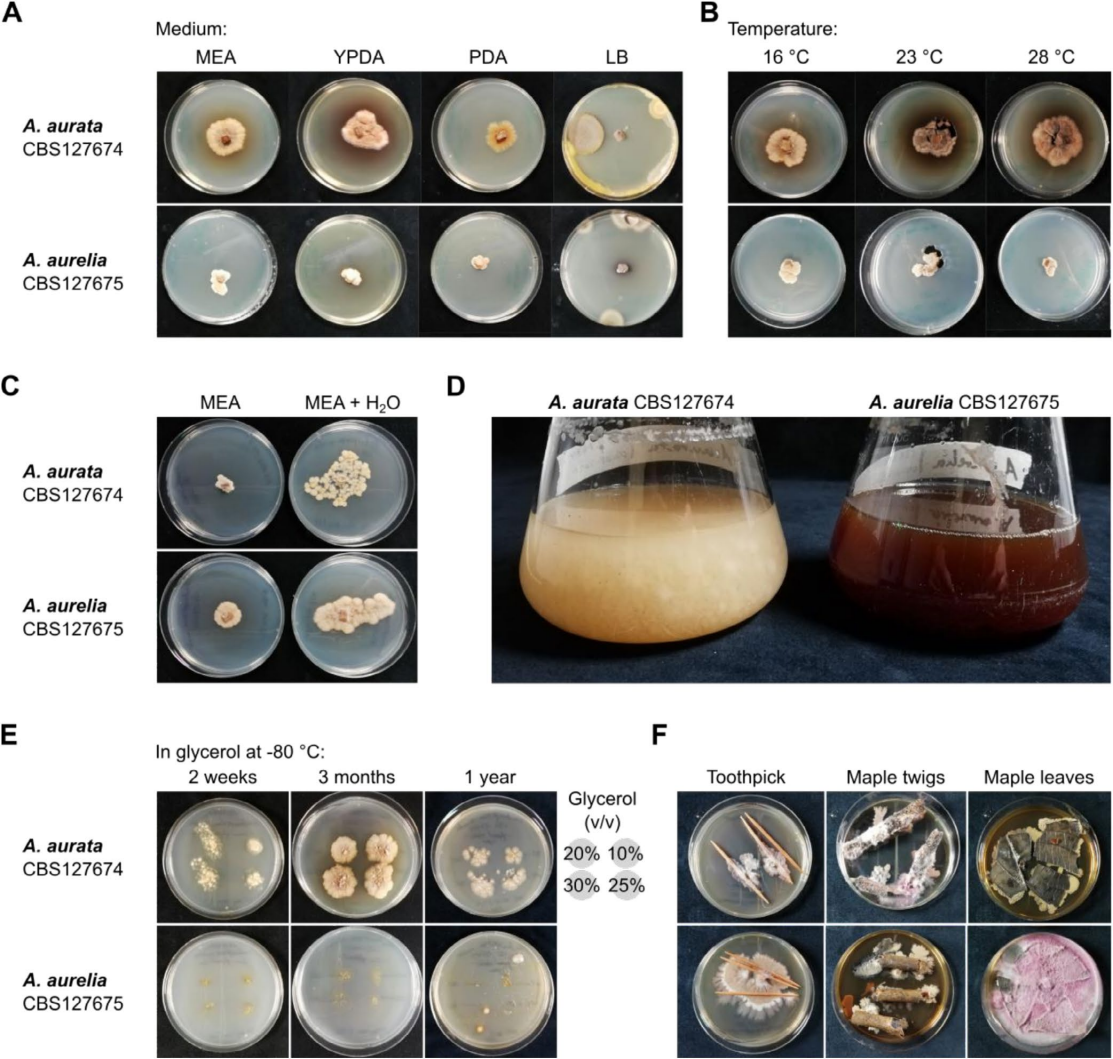
*Arachnopeziza aurata* and *A. aurelia* can be cultivated under standard in vitro growth conditions. The strains *Arachnopeziza aurata* CBS127674 and *A. aurelia* CBS127675 were cultivated on solid and liquid medium, inoculated using agar plugs containing mycelium. (A) *A. aurata* and *A. aurelia* were incubated on agar plates (from left to right, malt extract agar (MEA), yeast peptone dextrose agar (YPDA), potato dextrose agar (PDA), and lysogeny broth (LB)) at 23°C. Photos were taken 24 days after inoculation. (B) The fungi were grown on MEA plates at 16°C, 23°C, or 28°C. Photos were taken 24 days after inoculation. (C) *A. aurata* and *A. aurelia* were incubated on dry MEA plates (left) or by the addition of 200 μL of sterile H_2_O at 23°C. Photographs were taken 14 days after inoculation. (D) The fungi were grown in potato dextrose broth (PDB) at 80 rpm and 28°C. Photos taken 12 days after inoculation. (E) *A. aurata* and *A. aurelia* were incubated in PDB, and glycerol was added at final concentrations of 10% (v/v), 20% (v/v), 25% (v/v), and 30% (v/v), arranged as indicated on each plate; cultures were then flash‐frozen in liquid nitrogen and stored at −80°C. Cultures were recovered after 2 weeks, 3 months, and 1 year of storage by inoculating 50 μL of glycerol stock on MEA and incubation at 23°C for 14 days. (F) MEA plates were covered with (from left to right) sterile toothpicks, sterile maple twigs, or sterile maple leaf segments and inoculated with *A. aurata* or *A. aurelia* . The plates were incubated at 23°C, and photographs were taken after 3 months.

Effective and reproducible genetic modification protocols rely on in vitro cultivation methods (Lichius et al. 2020). We tested if *A. aurata* CBS127674 and *A. aurelia* CBS127675 can be cultivated on standard media used for the cultivation of fungi, that is, malt extract agar (MEA), potato dextrose agar (PDA), and yeast peptone dextrose agar (YPDA). Both fungi formed visible mycelium on the three media but did not grow on lysogeny broth (LB), a standard growth medium for bacteria (Figure 1A). Fungal growth was detected at 23°C and 28°C (Figure 1B) but was restricted at 37°C, and an increase in humidity by the addition of sterile water on PDA or MEA positively affected the colony size of both fungal species (Figure 1C). Both fungi also grew well in liquid medium (potato dextrose broth; PDB) at 28°C within 7 days of cultivation (Figure 1D). In addition, we were able to recover growing mycelium of *A. aurata* but not *A. aurelia* after flash‐freezing of liquid culture samples in glycerol at concentrations ranging from 10% to 30% after up to 1 year of storage at −80°C (Figure 1E). As *Arachnopeziza* species are wood‐ or plant litter‐decaying fungi (Ekanayaka 2019; Korf 1951), we also added sterilised toothpicks or pieces of twigs and leaves from a local maple tree to MEA. We found that *A. aurelia* specifically overgrew leaves but not wood, while *A. aurata* overgrew wood, but not leaves, after 3 months of cultivation (Figure 1F). Overall, we successfully cultivated two *Arachnopeziza* species in vitro using media commonly used for fungal propagation.

### 3.2 *Arachnopeziza* Species Have Compact Genomes

**FIGURE 2:**
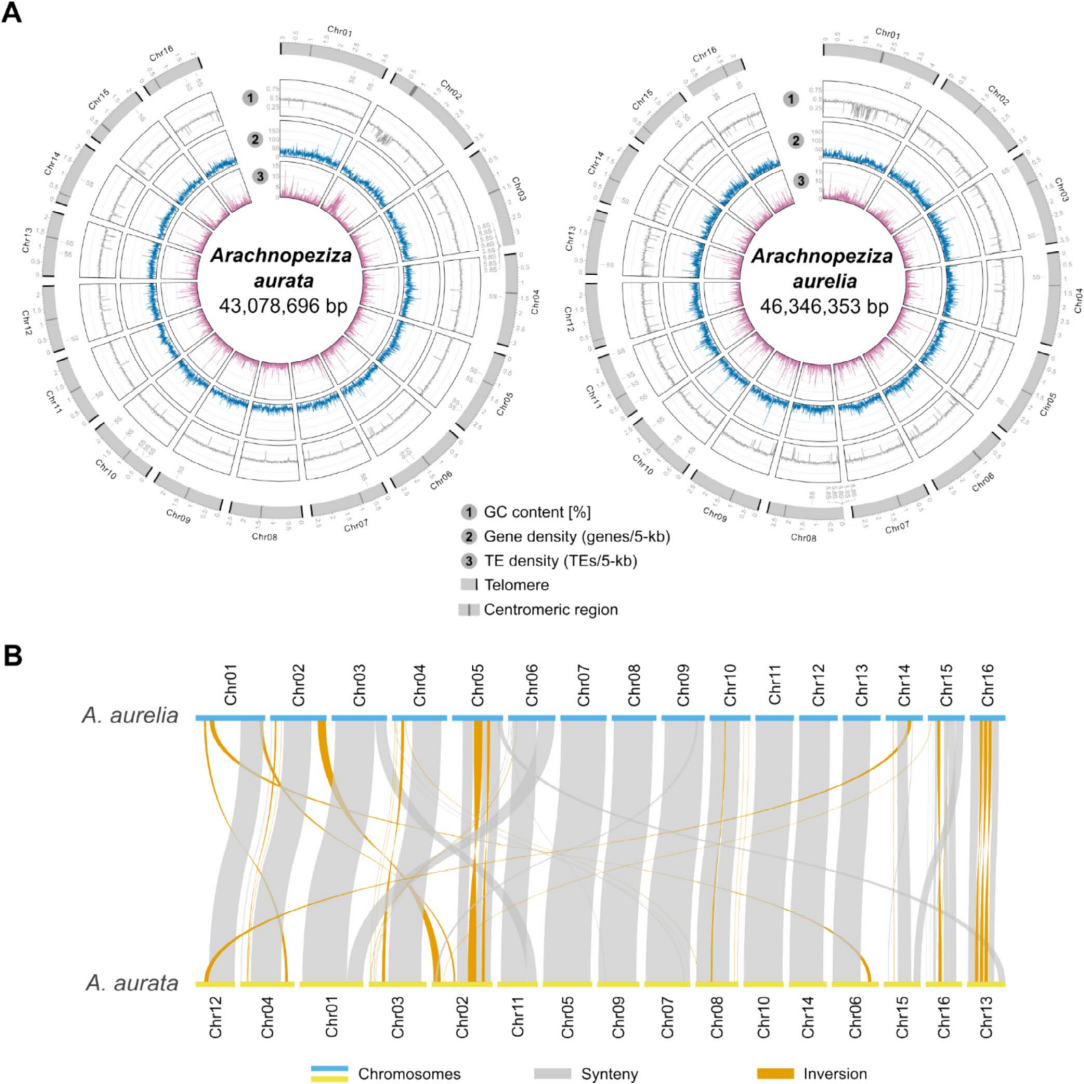
The chromosome‐scale genome assemblies for *Arachnopeziza aurata* and *A. aurelia* exhibit a high level of synteny. We generated genome assemblies for *A. aurata* CBS127674 and *A. aurelia* CBS127675 using MinION nanopore technology. (A) The circos plots display the genomic maps for *A. aurata* (left panel) and *A. aurelia* (right panel) and were generated with the R package circlize v0.4.10 (Gu et al. 2014). The outer tracks indicate the chromosomal map and the scale the position in million base pairs (Mbp); black rectangles denote telomeres and dark grey rectangles putative centromeres. We searched for potential candidate centromeric regions by identifying sliding windows covering between 30,000 and 150,000 bp where the gene content was zero or near zero and the TE content was at least 5 TEs per window. The tracks show GC content (1), gene density expressed as the number of genes per 5‐kb window (2), and TE density calculated as TEs per 5‐kb window (3) as line graphs. (B) The synteny plot shows the collinearity between the genome assemblies of *A. aurata* and *A. aurelia* and was generated with the R package RIdeogram v0.2.2 (Hao et al. 2020). The blue and yellow lines display the respective chromosomes, grey bars show blocks of synteny, and orange bars denote inversions.

To generate genomic resources for the fungal species *A. aurata* and *A. aurelia* , we obtained long sequence reads using MinION nanopore technology from in vitro‐cultured fungi for genome assembly. We merged the assemblies generated with three long‐read assemblers (Canu, Flye, and NextDenovo), yielding haploid genomes composed of 16 nuclear pseudo‐chromosomes for both species (Table 1 and Figure 2A). We then used teloclip (https://github.com/Adamtaranto/teloclip) to retrieve nanopore reads at both ends of the assembled sequences containing telomeric repeats (5′‐TTAGGG‐3′) and thus recovered 31 of the 32 telomeric ends in the genome assembly of *A. aurata* and 30 out of 32 telomeres in the case of *A. aurelia* (Figure 2A). Additionally, we determined putative centromeric regions of the 16 pseudo‐chromosomes per species. The final assemblies contained 43,078,696 base pairs (bp) in the case of *A. aurata* and 46,346,353 bp for *A. aurelia* with N50 values of 2,695,373 and 2,924,875 bp, respectively (Table 1). To confirm the identity of both assemblies, we extracted the 5.8S, 18S, 28S nuclear ribosomal DNA (nrDNA) and the corresponding internal transcribed spacer (ITS) sequences and found that they were 99% identical to the respective nrDNA sequences deposited in GenBank (accessions MH864617.1, MH876055.1, MH864618.1, and MH876056.1; see Files S1 and S2 for alignments). We also used Benchmarking Universal Single‐Copy Orthologs (BUSCO) to assess the completeness of the two genome assemblies and found 99.0% of the 1706 core ascomycete genes to be present as single copies in the two assemblies (Table 1). By comparison, the available draft assembly of *A. araneosa* (Johnston et al. 2019) was generated using short‐read sequencing from an environmental sample and, therefore, is highly fragmented, as, for example, indicated by a low N50 value of 210,669 bp (more than 10 times lower than for *A. aurata* and *A. aurelia* ; Table 1). Thus, the assemblies generated in the current work comprise significantly improved resources for *Arachnopeziza* species. The genomes of the two species *A. aurata* and *A. aurelia* exhibited overall high levels of collinearity, although structural rearrangements could be observed for some chromosomes (Figure 2B). For instance, *A. aurelia* chromosome 6 was collinear in part with *A. aurata* chromosomes 1 and 11.

**TABLE 1:** Genome assembly statistics for *A. aurata* and *A. aurelia* compared to *A. araneosa*.

Feature	*A. araneosa* ^a^	*A. aurata*	*A. aurelia*
Genome assembly (bp)	40,453,245	43,078,696	46,346,353
N count	3,840	0	0
Estimated size (k‐mer) (bp)	—	43,965,082	45,594,330
Scaffolds/contigs	1,498	16	16
Largest sequence (bp)	1,164,973	3,758,135	4,390,527
Average length (bp)	27,004.8	2,692,418.5	2,896,647.1
GC content (%)	45.63	44.49	43.26
N50	210,669	2,695,373	2,924,875
N90	46,250	2,227,591	2,344,259
CRAQ^b^			
Sequence covered (%)	—	99.99	99.99
R‐AQI	—	92.84	93.32
S‐AQI	—	100.00	100.00
BUSCO^c^			
Complete single‐copy	1,690 (99.1%)	1,689 (99.0%)	1,688 (99.0%)
Duplicated	2 (0.1%)	3 (0.2%)	2 (0.1%)
Fragmented	12 (0.7%)	13 (0.8%)	15 (0.9%)
Missing	2 (0.1%)	1 (0.0%)	1 (0.0%)
Interspersed repeats (%)^d^	0.83	2.63	3.93
SINE retroelements (%)	0.00	0.01	0.00
LINE retroelements (%)	0.00	0.00	0.00
LTR elements (%)	0.72	2.36	2.50
DNA transposons (%)	0.11	0.20	0.10
Other/Unclassified (%)	0.01	0.07	1.33

^a^ Strain D781; retrieved from NCBI genomes database at accession ASM398885v1 (Johnston et al. 2019).

^b^ Determined using CRAQ v1.0.9 (Li et al. 2023); R‐AQI, regional assembly quality index; S‐AQI, overall assembly quality index. AQI values > 90 are considered reference quality.

^c^ Ran with 1706 core ascomycete genes from the database ascomycota_odb10 using compleasm version 0.2.6 (Huang and Li 2023; Simão et al. 2015).

^d^ Database was calculated with an EDTA2‐TE trimmer pipeline (Qian et al. 2025).

In addition, we assembled the mitochondrial genomes of both species. The mitochondrial genomes had a size of 28,111 bp ( *A. aurata* ) and 28,095 bp ( *A. aurelia* ), respectively. By comparison, currently available fungal mitogenomes range from 12 kb in *Rozella allomycis* (James et al. 2013) to 531 kb in *Morchella crassipes* (Liu et al. 2020). All components of the mitochondrial respiratory complexes I, III, and IV, both small and large ribosomal RNAs *rns* and *rnl*, the gene coding for ribosomal protein S3 (*rps3*), and the ribonucleoprotein RNA *rnpB*, which are typically found in fungal mitochondrial genomes (Aguileta et al. 2014; Seif et al. 2005), were present. The sole exception was the ATP synthase component *atp8*, which we did not detect in either of the two mitochondrial genomes (Figure S1). Loss of *atp8* (along with *atp9*) in the mitochondrial genomes due to migration to the nuclear genome has been observed for other fungi, such as *Stemphylium lycopersici* (Franco et al. 2017). Similarly, we found an orthologue of fungal *atp8* in each of the nuclear genomes of *A. aurata* and *A. aurelia* , encoded by *AAURAT_014873‐RA* on Chr15 and *AAUREL_012968‐RA* on Chr14, respectively. Thus, *atp8* migrated to the nuclear genome in the two *Arachnopeziza* species. By contrast, *atp8* is present in the mitochondrial genomes of powdery mildew fungi such as *Erysiphe necator* (Zaccaron et al. 2021), *B. graminis* f. sp. *tritici*, *E. pisi*, and *Golovinomyces cichoracearum* (Zaccaron and Stergiopoulos 2021).

**FIGURE 3:**
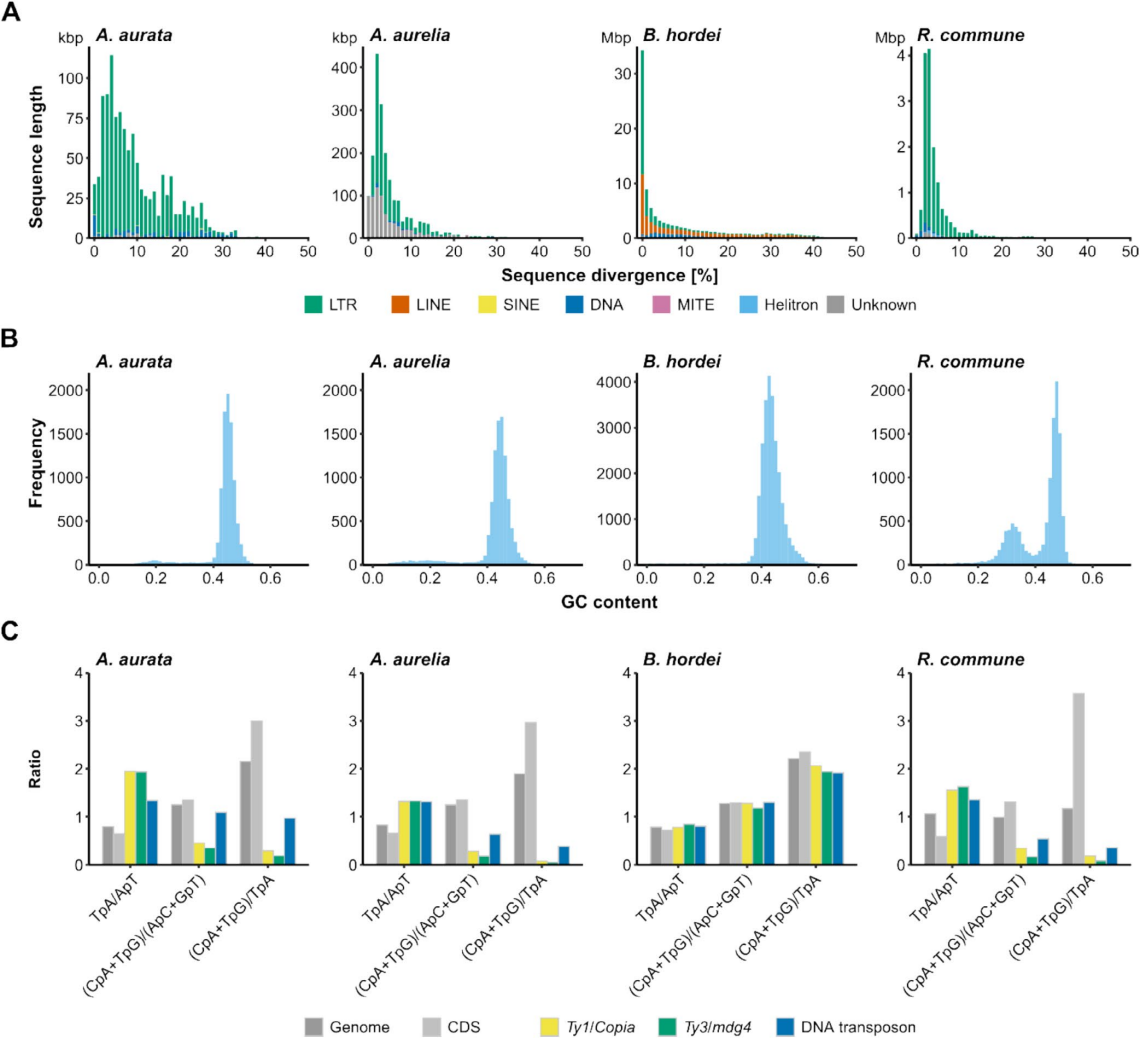
The genomes of *Arachnopeziza aurata* and *A. aurelia* show signs of TE degeneration. We compared the repetitive content of the genome assemblies of *A. aurata* CBS127674 and *A. aurelia* CBS127675 with the assemblies of the barley powdery mildew *Blumeria hordei* DH14 (Frantzeskakis et al. 2018) and the barley leaf scald disease pathogen *Rhynchosporium commune* UK7 (Penselin et al. 2016). Panels are displayed in this order from left to right. (A) The stacked bar graphs show the sequence divergence of TE subclasses in percent (x‐axis) and the cumulative length of sequence at the respective level of divergence in base pairs (kbp or Mbp, as indicated; y‐axis). The bars are coloured according to TE class: Long terminal repeat (LTR), green; long interspersed nuclear element (LINE), orange; short interspersed nuclear element (SINE), yellow; DNA transposons, blue; miniature inverted‐repeat transposable element (MITE), purple; DNA Helitron, light blue; unknown or unclassified, grey. (B) The histograms display the distribution of GC content (ratio, x‐axis) determined in genome‐wide 5‐kb windows; the y‐axis shows the frequency of the respective content bin. (C) The bar graphs show the ratios of repetitive sequence dinucleotide analysis genome‐wide (dark grey), for coding sequences (CDS; light grey), *Ty1*/*Copia* LTR elements (yellow), *Ty3*/*mdg4* LTR elements (orange), and DNA transposons (blue). The respective RIP index represented by dinucleotide ratios is indicated on the x‐axis, and the y‐axis displays the respective ratio.

Next, we used EDTA2 and TEtrimmer (Ou et al. 2019; Qian et al. 2025) to annotate transposable elements (TEs) and repeats in the genomes of *A. aurata* and *A. aurelia* . The species exhibited a repeat content of 2.6% and 3.9% (Table 1), respectively, which is well below the values of most powdery mildew fungi, which typically have repeat contents of 40%–80% (Kusch, Qian, et al. 2024). The transposon space was dominated by long terminal repeat (LTR) retroelements, while long interspersed nuclear elements (LINEs) were absent in both genomes (Table 1). TEs were mostly located in gene‐sparse regions with low GC content (Figure 2A). In contrast to powdery mildew fungi, whose genomes often show signatures of recent or ongoing TE bursts and no signs of the fungal TE defence mechanism repeat‐induced point mutation (RIP) (Frantzeskakis et al. 2018, 2019; Kusch, Qian, et al. 2024), the TEs in the *Arachnopeziza* genomes had a peak sequence divergence of 2%–10%, suggestive of evolutionary older transposition events, and exhibited weak but detectable bipartite GC content distribution, indicative of the RIP defence mechanism (Figure 3A,B). We performed RIP index analysis using dinucleotide ratios calculated by RIPCAL (Hane and Oliver 2008) and compared the *Arachnopeziza* genome assemblies with that of the barley powdery mildew pathogen *Blumeria hordei* DH14, which does not exhibit RIP signatures (Frantzeskakis et al. 2018), and *Rhynchosporium commune*, an example of a genome exhibiting enrichment of AT‐rich sequences in repetitive regions, indicative of RIP activity (Frantzeskakis et al. 2019) (Figure 3C and Figure S2). In the prototypical case of the model fungus *Neurospora crassa*, sequences with a TpA/ApT index above 0.89 and a (CpA + TpG)/(ApC + GpT) index below 1.03 suggest AT enrichment due to RIP (Margolin et al. 1998). We found the TpA/ApT indices of *Ty1*/*Copia* and *Ty3*/*mdg4* LTR retroelements to be above 1.9 in the case of *A. aurata* and above 1.3 in the case of *A. aurelia* , while the index was above 1.3 for DNA transposons in both species (Figure 3C). By comparison, the TpA/ApT ratio was around 0.6 for coding sequences (CDSs) and around 0.8 genome‐wide. On the other hand, the (CpA + TpG)/(ApC + GpT) indices were below 0.5 for both types of LTR retrotransposons in both species, while DNA elements had an index of 0.63 for *A. aurelia* and 1.09 for *A. aurata* ; the indices for CDSs were 1.35 and genome‐wide 1.25 (Figure 3C). The difference between CDSs and the analysed TEs was even more pronounced for the (CpA + TpG)/TpA index. The pattern of markedly increased TpA/ApT in combination with decreased (CpA + TpG)/(ApC + GpT) and (CpA + TpG)/TpA indices in DNA transposons and retroelements was similar in *R. commune*, while these indices exhibited no discernible difference for all analysed features in *B. hordei*, reflecting the observed presence and absence, respectively, of RIP in these organisms (Frantzeskakis et al. 2018, 2019). Furthermore, we detected one orthologue each of the key components of RIP in fungi, that is, *Masc1*, *Masc2*, *Rid‐1*, and *Dim‐2* (GenBank accession numbers AAC49849.1, AAC03766.1, XP_011392925.1, and XP_959891.1, respectively) (Gladyshev 2017), in *A. aurata* (E values 5e‐63, 1e‐70, 8e‐89, and 0.0, respectively), and one orthologue of *Rid‐1* (E value 2e‐78) and *Dim‐2* (E value 0.0) each in *A. aurelia* (Files S3 and S4), while these genes are known to be absent in powdery mildew fungi (Frantzeskakis et al. 2019; Kusch, Qian, et al. 2024; Spanu et al. 2010). Overall, the genome assemblies of both *A. aurata* and *A. aurelia* showed an enrichment of AT dinucleotide signatures in LTR retroelements and, to a different degree, in DNA transposons, in combination with the presence of key RIP components, indicative of a functional RIP defence mechanism in these fungi.

### 3.3 *Arachnopeziza* Species Exhibit the Genomic Signatures of a Saprobic Fungus With Plant Cell Wall‐Degrading Ability

We used global RNA sequencing data obtained from mycelium grown in liquid culture for both species (Figure 1D) to discover and annotate coding genes using BRAKER3 (Bruna et al. 2023; Gabriel et al. 2021, 2023). Overall, we predicted 15,452 genes in *A. aurata* and 13,437 genes in *A. aurelia* (Table 2), which are higher than the average gene number of ~11,000 in ascomycetes (Mohanta and Bae 2015).

**TABLE 2:** Gene annotation of *A. aurata* and *A. aurelia* .

Feature	*A. aurata*	*A. aurelia*
Number of coding genes^a^	15,452	13,437
Putatively secreted proteins^b^	1233	1109
Putative effectors^c^	536	433
Predicted CAZymes^d^	618	503
Contains protein family (PFAM) domain	12,360	10,505
Contains InterPro Superfamily domain	8,049	6,831
Secondary metabolism component^e^	69	73
Non‐ribosomal peptide synthetases (NRPS)	14	25
Polyketide synthases (PKS)	31	22
Fungal ribosomally synthesised and post‐translationally modified peptide (RiPP)‐like	18	21
Terpene biosynthesis	6	4
Indole biosynthesis	0	1

^a^ Genes predicted using BRAKER3 (Bruna et al. 2023; Gabriel et al. 2021, 2023).

^b^ Secretion signals were predicted using SignalP5.0 (Almagro Armenteros et al. 2019).

^c^ Predicted using EffectorP3.0 (Sperschneider and Dodds 2021).

^d^ CAZymes were predicted using dbCAN3 (Zheng et al. 2023).

^e^ Predicted using antiSMASH v7.1.0 (Blin et al. 2023; Medema et al. 2011).

**FIGURE 4:**
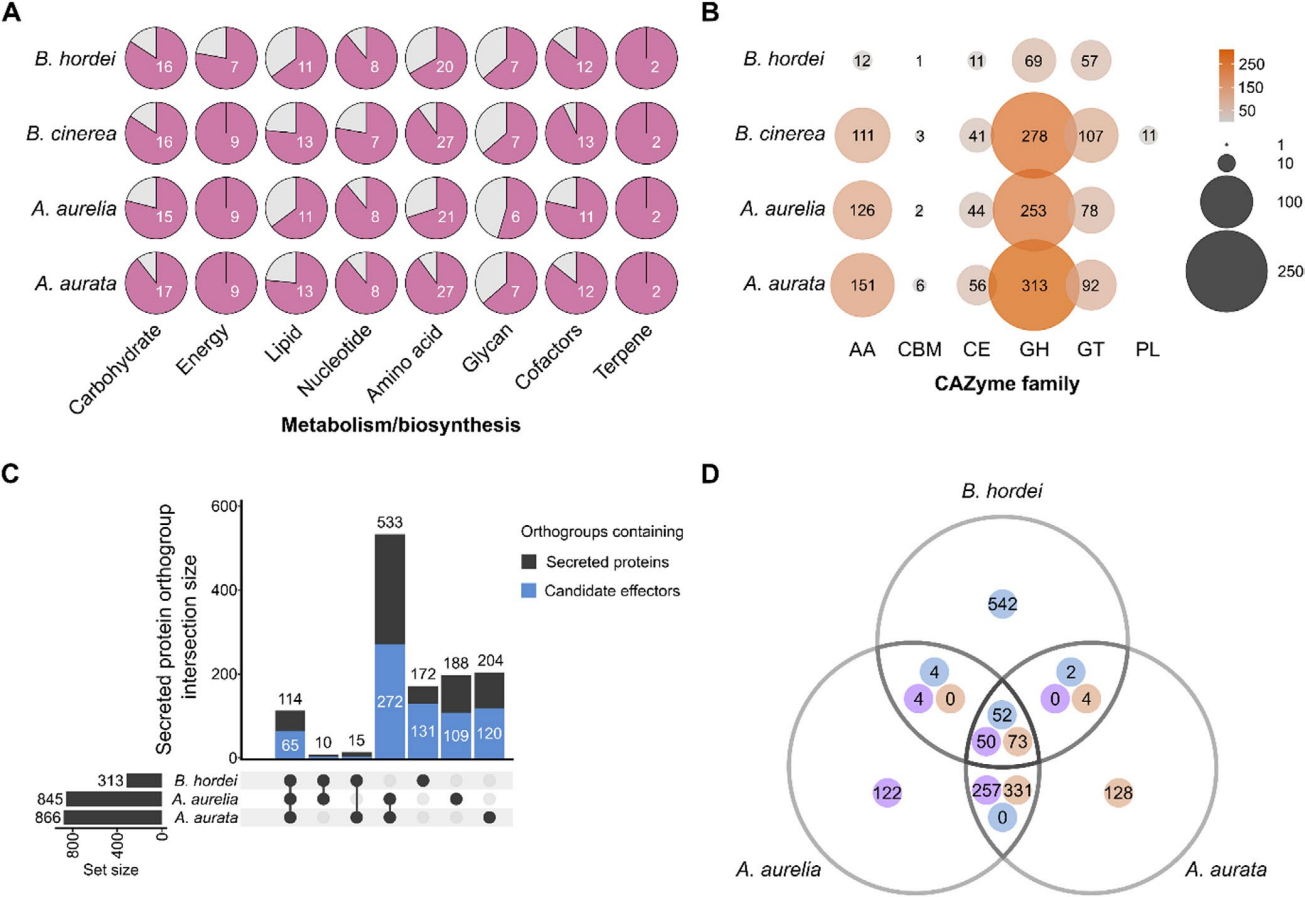
*Arachnopeziza aurata* and *A. aurelia* harbour genes enabling a saprobic lifestyle and plant cell wall degradation, and possess candidate effectors that may facilitate host interactions. (A) Primary metabolism modules were annotated using GhostKOALA to identify components and KEGG Mapper to reconstruct pathways via https://www.kegg.jp/ (accessed 04/2025) in *A. aurata* , *A. aurelia* , *B. cinerea* B05.10 (Van Kan et al. 2017), and *B. hordei* DH14 (Frantzeskakis et al. 2018). The pie charts indicate the number of complete pathways in the respective categories (x‐axis), that is, carbohydrate metabolism (19 pathways in fungi), energy metabolism (9 pathways), lipid metabolism (17 pathways), nucleotide metabolism (9 pathways), amino acid metabolism (30 pathways), glycan metabolism (11 pathways), cofactor metabolism (14 pathways), and terpene biosynthesis (2 pathways). (B) We used dbCAN3 (Zheng et al. 2023) to annotate putative carbohydrate‐active enzymes in *A. aurata* , *A. aurelia* , *B. cinerea* B05.10 (Van Kan et al. 2017), and *B. hordei* DH14 (Frantzeskakis et al. 2018). The dot plot indicates the number of genes annotated to encode accessory activity (AA) enzymes, carbohydrate‐binding motif (CBM)‐containing proteins, carbohydrate esterases (CE), glycosyl hydrolases (GH), glycosyl transferases (GT), and polysaccharide lyases (PL). The size of the dots and their shade of orange are proportional to the number of predicted genes in each category; the number is also written on each dot. (C) The UpSet plot shows the overlap of orthologous groups of putative secreted proteins from *B. hordei*, *A. aurata* , and *A. aurelia* (Table 2). Signal peptides for secretion were predicted using SignalP5.0 (Almagro Armenteros et al. 2019), and orthologous groups (orthogroups) were assigned with OrthoFinder v2.5.5 (Emms and Kelly 2019). The blue portion of the bars indicates orthogroups containing at least one candidate effector protein as determined by EffectorP3.0 (Sperschneider and Dodds 2021). The sets corresponding to each bar are indicated below the bar chart by black dots. (D) The Venn diagram illustrates the orthologous overlap of candidate effector proteins from *B. hordei* (blue dot), *A. aurata* (orange dot), and *A. aurelia* (purple dot), as determined by EffectorP3.0.

We used Kyoto Encyclopedia of Genes and Genomes (KEGG) Mapper (https://www.kegg.jp/) to reconstruct the primary metabolism pathways in *A. aurata* and *A. aurelia* (Figure 4A and Tables S2–S4). We recovered most metabolic pathways related to carbohydrate, energy, lipid, amino acid, and cofactor metabolism in *A. aurata* . In the case of *A. aurelia* , several components of the amino acid metabolism were not found, specifically of the branched‐chain amino acid metabolism pathways related to valine, isoleucine, and leucine biosynthesis. Nonetheless, both fungi display a comparable primary metabolism profile to the fungal plant pathogen *Botrytis cinerea* (Figure 4A) and do not show the losses in sulfur assimilation and thiamine biosynthesis reported for the obligate biotrophic powdery mildew fungus *B. hordei* (Frantzeskakis et al. 2018; Spanu et al. 2010; Sabelleck, Freh, et al. 2025) and other powdery mildew species (Frantzeskakis et al. 2019).

Further, 618 and 503 genes were predicted to encode carbohydrate‐active enzymes (CAZymes) according to a dbCAN3 search (Zheng et al. 2023) (Table 2, Tables S2 and S3). Similar to the necrotroph *B. cinerea* , which is capable of digesting plant‐derived carbohydrates, including those present in cell walls (Kubicek et al. 2014), both *A. aurata* and *A. aurelia* possess large numbers of putative glycosyl hydrolases, glycosyl transferases, carbohydrate esterases, and auxiliary enzymes (Figure 4B). This contrasts with the powdery mildew pathogen *B. hordei*, which exhibits a reduced CAZyme complement, particularly of glycosyl hydrolases and auxiliary enzymes (Frantzeskakis et al. 2018). Different from *B. cinerea* , the two *Arachnopeziza* species do not seem to harbour polysaccharide lyases, which cleave uronic acid‐containing polysaccharide chains and include fungal pectin lyases (Lombard et al. 2010). The pectin lyase family appears to be expanded in plant pathogens but is often absent in saprobic fungi (Karlsson et al. 2015), consistent with the non‐pathogenic lifestyle of the *Arachnopeziza* species.

According to analysis by SignalP5.0 (Almagro Armenteros et al. 2019), we found that 1233 *A. aurata* and 1109 *A. aurelia* genes are predicted to encode secreted proteins, of which 536 and 433 are putative effector proteins according to EffectorP3.0 (Sperschneider and Dodds 2021) (Table 2). Of these, 266 ( *A. aurata* ) and 231 ( *A. aurelia* ) have unknown functions (no protein families (PFAM) domain detected; Tables S2 and S3). The most common functional predictions for the remaining effector candidates are related to carbohydrate‐binding or modification, peptidase activity, and the presence of a common fungal extracellular membrane (CFEM) domain. We compared the *Arachnopeziza* candidate effectors with annotated candidate secreted effector proteins (CSEPs) of *B. hordei* DH14 (Frantzeskakis et al. 2018) and added putative secreted proteins predicted to be effectors according to an EffectorP3.0 search (Sperschneider and Dodds 2021), totalling 600 *B. hordei* candidate effectors. We found that 139 orthologous groups of proteins (orthogroups) are shared between *B. hordei*, *A. aurata* , and *A. aurelia* candidate secreted proteins, while 533 orthogroups are exclusively shared between the two *Arachnopeziza* species (Figure 4C and Table S5). Of the 139 shared orthogroups, 65 contained at least one candidate effector. Among these, 52 *B. hordei* candidate effectors were orthologous with 73 *A. aurata* and 50 with *wiA. aurelia* candidate effectors (Figure 4D). On the other hand, 257 *A. aurelia* effectors were orthologous with 331 *A. aurata* candidate effectors, while these were absent in *B. hordei*. Likewise, most *B. hordei* candidate effectors (542) have no orthologue in either *Arachnopeziza* species, including the absence of seven powdery mildew core effectors (Sabelleck, Deb et al. 2025). The family of ribonuclease‐like effectors expressed in haustoria (RALPHs) is ubiquitously present in powdery mildew fungi (Frantzeskakis et al. 2019) and particularly abundant in cereal powdery mildew fungi (Pedersen et al. 2012; Spanu 2017). We searched the putative secretome of *A. aurata* and *A. aurelia* for the presence of domains of the ribonuclease superfamily (IPR016191) and found AAUREL_003350‐RA as the only case of an effector candidate with this domain. Unlike RALPH effectors of powdery mildew fungi, AAUREL_003350‐RA displays sequence conservation of the residues required for ribonuclease activity in RNase T1, suggesting that it is a catalytically active ribonuclease (Figure S3 and File S5 for alignment). Additional searches for PFAM domains (PF00445, PF06479) and InterPro domains (IPR036430) from the ribonuclease T2‐like superfamily identified three *A. aurata* and three additional *A. aurelia* secreted proteins that were assigned at least one of these domain accessions (Table S6). However, sequence similarity analysis indicated that six of these ribonuclease‐like proteins are more similar to fungal ribonuclease T1/T2/Trv proteins than RNase‐like effectors of powdery mildew fungi (Figure S4; see File S6 for alignment). One (AAURAT_002659) is an orthologue of the endoplasmic reticulum membrane‐localised serine/threonine kinase inositol‐requiring protein 1 (IRE1), which contains a ribonuclease domain and is involved in the unfolded protein (UPR) stress response (Credle et al. 2005). Overall, this analysis suggests that *Arachnopeziza* fungi do not harbour RNase‐like effectors.

Fungi biosynthesize a large range of secondary metabolites to communicate and compete with other organisms, for protection from abiotic stress, and to regulate their development (Keller 2019; Nguyen et al. 2023). We used antiSMASH v7.1.0 (Blin et al. 2023; Medema et al. 2011) to discover components of secondary metabolism in *A. aurata* and *A. aurelia* . Both genomes encode around 70 putative components of secondary metabolite biosynthesis, including non‐ribosomal peptide synthetases (NRPS), polyketide synthases (PKS), and fungal ribosomally synthesised and post‐translationally modified peptide‐like (fungal‐RiPP‐like) proteins (Table 2 and Tables S2 and S3). Several of the genes encoding these components are located close to each other and may constitute clusters involved in secondary metabolite biosynthesis (Figure S5), a common feature found in fungi (Nguyen et al. 2023; Robey et al. 2021). By comparison, we found only eight candidate secondary metabolite biosynthetic genes in *B. hordei*, in line with observed gene losses in this fungus in the context of its obligate biotrophic lifestyle (Frantzeskakis et al. 2018; Spanu et al. 2010).

Taken together, we observed that the two *Arachnopeziza* species possess the genetic components required for a saprobic lifestyle, including the ability to generate secondary metabolites, possibly to govern microbial competition, communication, and fungal development. The large complement of CAZymes similar to that of the necrotrophic plant pathogen *B. cinerea* , which devours plant biomass, including cell walls, points to the ability to degrade plant cell wall components such as cellulose and lignin. Unexpectedly, we identified more than 400 effector candidates in the two fungi, suggesting that *Arachnopeziza* species are capable of interacting with unknown plant hosts and/or microbes.

### 3.4 *A. aurata* and *A. aurelia* Are Sensitive to Common Fungicides and Antibiotics

**FIGURE 5:**
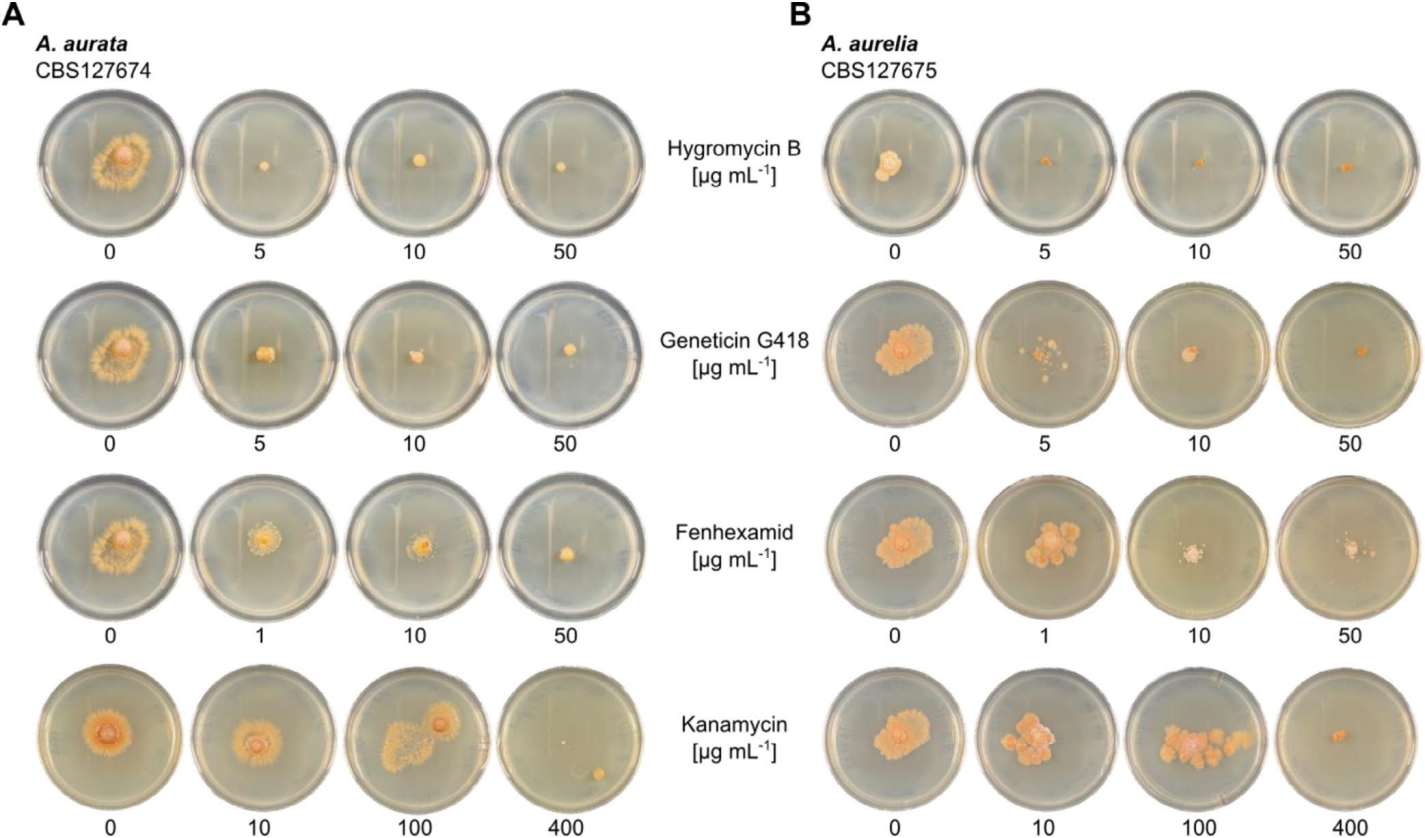
*Arachnopeziza aurata* and *A. aurelia* are inhibited by common fungicides. The strains (A) *Arachnopeziza aurata* CBS127674 and (B) *A. aurelia* CBS127675 were cultivated on potato dextrose agar (PDA) containing (top to bottom) hygromycin B, geneticin G418, fenhexamid, or kanamycin at the indicated concentrations. The plates were incubated at 23°C; photographs were taken 16 days after inoculation. Additional fungicides and antibiotics, and the full set of tested concentrations for kanamycin and fenhexamid, are shown in Figure S6. Note that the control pictures for the 0 μg mL^−1^ negative control are identical if the respective fungicides/antibiotics were tested in the same experiment.

Generating genetically modified microbes requires efficient selection markers to isolate successfully modified individuals. Antibiotic and fungicide resistance are frequently used features that serve as selection markers in microbes. We used a panel of antibiotics and fungicides commonly deployed for the selection of transformants and cultivated *A. aurata* and *A. aurelia* on PDA containing these antimicrobial compounds at varying concentrations. The fungicides hygromycin B and fenhexamid, and the antibiotic geneticin G418 exhibited growth‐inhibiting activity starting at concentrations of 5–10 μg mL^−1^ and fully suppressed the growth of both fungi at 10–50 μg mL^−1^ (Figure 5). In addition, streptomycin showed some inhibitory capacity against *A. aurata* at 400 μg mL^−1^ but not against *A. aurelia* (Figure S6). Further, kanamycin had inhibiting activity at 400 μg mL^−1^, while none of the other antibiotics affected the growth of the two *Arachnopeziza* species at the tested concentrations (Figure 5 and Figure S6). Hence, hygromycin B, fenhexamid, and geneticin qualify as effective selection markers for *A. aurata* and *A. aurelia* .

### 3.5 Protoplast‐Mediated Transformation Confers Hygromycin Resistance and Fluorescent Protein Expression in *A. aurata*

**FIGURE 6:**
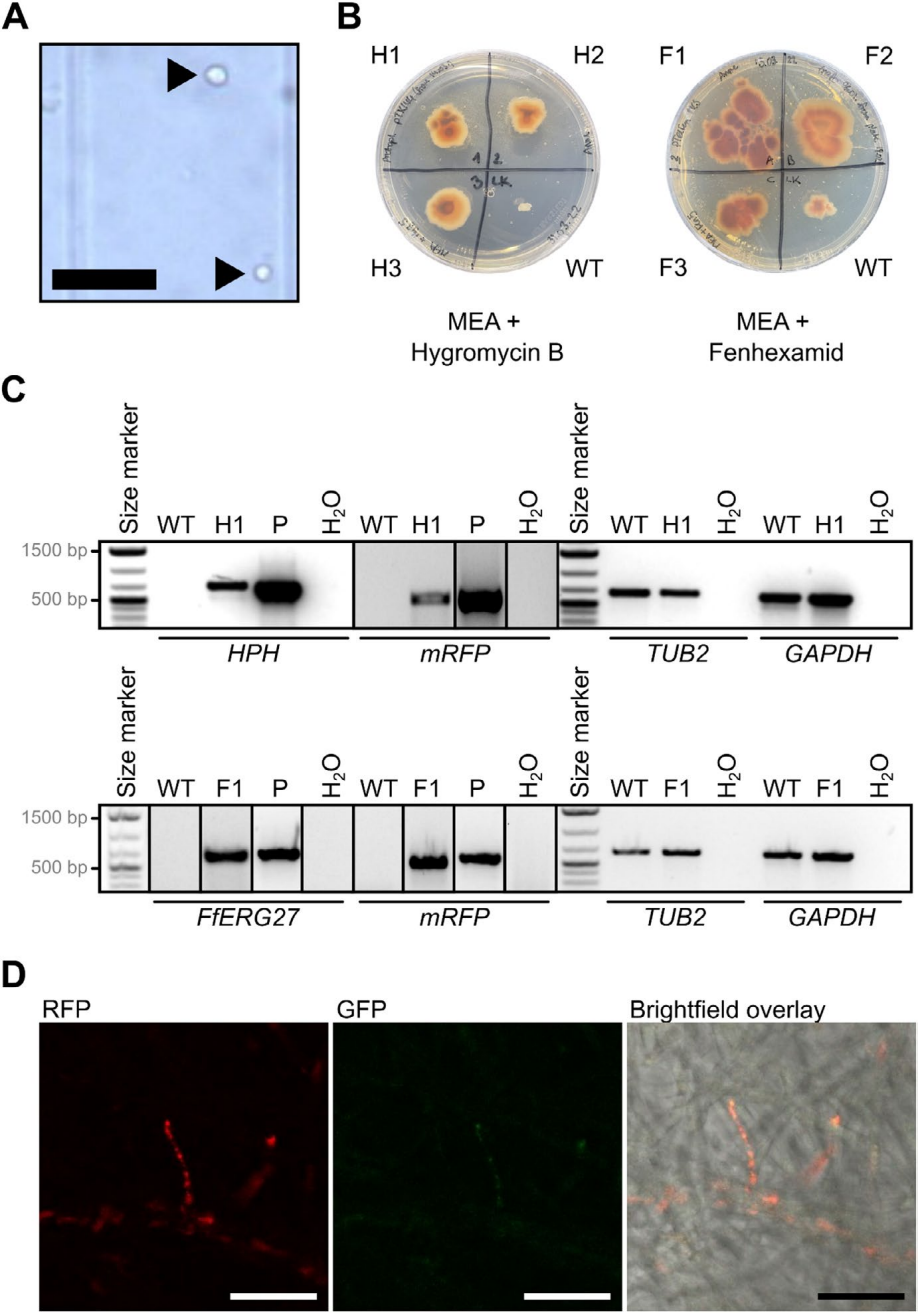
Transgenes conferring fungicide resistance and fluorescence can be expressed in *Arachnopeziza aurata*. *A. aurata* CBS127674 was genetically modified using a modified PEG‐mediated protoplast transformation protocol (Leisen et al. 2020; Wegner et al. 2022). (A) Brightfield micrograph of *A. aurata* protoplasts (arrows); scale bar: 50 μm. (B) Strains were incubated on MEA with 5 μg mL^−1^ hygromycin B (left) or fenhexamid (right) at 23°C. Transgenic strains are denoted H1‐H3 (pTK144; hygromycin resistance) and F1‐F3 (pTelFen, fenhexamid resistance). Photographs taken 16 days after incubation. (C) Genotyping PCRs for transgenic strains H1 and F1. The transgenes were: *HPH*, hygromycin resistance gene; *mRFP*, red fluorescent protein gene; *FfERG27*, fenhexamid resistance allele of *Fusarium fujikuroi*. Two endogenous *A. aurata* genes were genotyped as control, that is, *TUB2*, *A. aurata* tubulin 2‐coding gene; *GAPDH*, *A. aurata* glyceraldehyde 3‐phosphate dehydrogenase‐coding gene. Oligonucleotides are listed in Table S1; original gels displayed in Figure S7. Expected amplicon sizes were: *HPH*, 692 bp; *mRFP*, 591 bp; *FfERG27*, 648 bp; *TUB2*, 654 bp; *GAPDH*, 632 bp. P, positive control plasmids pTK144 (upper panel) and pTelFen (lower panel); H_2_O, no template negative control. Size marker, 1 kb plus (Invitrogen‐Thermo Fisher, Waltham, MA, USA). (D) Confocal laser scanning microscopy of mycelium obtained from strain H1. Left, monomeric red fluorescence protein (mRFP) excitation at 561 nm and detection at 595–645 nm; middle, green fluorescence protein (GFP) excitation at 488 nm and detection at 505–555 nm (autofluorescence control); right, brightfield‐mRFP channel overlay. Micrographs were taken with an SP8 confocal laser scanning microscope (Leica Microsystems GmbH, Wetzlar, Germany) using the LAS‐X software. Scale bar: 25 μm.

Next, we aimed to determine if a common fungal transformation method can achieve genetic modification of *Arachnopeziza* species. We used a modified polyethylene glycol (PEG)‐mediated protoplast transformation protocol established previously for *Magnaporthe oryzae* (Leisen et al. 2020; Wegner et al. 2022). After successful isolation of fungal protoplasts from liquid culture of *A. aurata* (Figure 6A), we used previously established vectors (pTelFen carrying the fenhexamid resistance allele of *Fusarium fujikuroi*, *FfERG27*) (Cohrs et al. 2017; Wegner et al. 2022), and pTK144 conferring hygromycin resistance, and both harbouring the monomeric red fluorescent protein (mRFP)‐coding gene for protoplast transformation. We obtained one fenhexamid‐ and one hygromycin B‐resistant *A. aurata* line and confirmed the presence of the *FfERG27* (fenhexamid resistance allele), *HPH* (hygromycin resistance gene), and *mRFP* genes via genotyping by PCR (Figure 6B,C). We further confirmed the presence of the mRFP protein in the hyphae of some of the transformed lines by the detection of characteristic fluorescent signals via confocal laser scanning microscopy (Figure 6D). These experiments demonstrate that genes can be heterologously expressed in *A. aurata* using protoplast transformation.

## 4 Discussion

The closest known extant relatives of the powdery mildew fungi belong to the *Arachnopezizaceae*, a family of wood‐ and litter‐decaying saprotrophic fungi (Ekanayaka 2019; Johnston et al. 2019; Korf 1951; Vaghefi et al. 2022). In contrast to the obligate biotrophic powdery mildews, *Arachnopeziza* grows in vitro using common media and cultivation procedures (Figure 1; Kosonen et al. 2020), which implies that genetic modification and selection protocols may be feasible. We noted growth limitations for both fungi concerning temperature and carbon source (Figure 1A,F). In these instances, we often observed contaminations where *Arachnopeziza* did not grow, which, however, can be explained by the long incubation periods of over 2 weeks and up to 3 months, and that did not confound these experiments. In this work, we established high‐quality genomic resources for the two species *A. aurata* and *A. aurelia* , including telomere‐to‐telomere genome assemblies (Figure 2) and coding gene and transposon annotations (Tables 1 and 2 and Tables S2 and S3). We demonstrated that *A. aurata* is amenable to protoplast‐based genetic modification by conferring transgene‐mediated hygromycin resistance and red fluorescence (Figures 5 and 6), suggesting that *A. aurata* may represent a useful tool for the genetic study of the obligate biotrophic lifestyle in powdery mildew fungi.

Fungi exhibit various lifestyles depending on the ecological niche they occupy (Lowe and Howlett 2012). Saprotrophic fungi can decompose organic matter, including complex polysaccharides occurring in wood, bark, and plant litter. Obligate biotrophic fungi obtain carbohydrates and inorganic nutrients exclusively from a living host, while necrotrophic fungi kill and decompose their host while feeding on it. Obligate biotrophic plant pathogens, including powdery mildew fungi, rely on their host to obtain certain metabolites, as they have lost certain biosynthetic pathways (Spanu 2012). Interestingly, saprotrophic fungi can undergo lifestyle transitions towards necrotrophy or facultative biotrophy under certain conditions and with compatible hosts (Hill et al. 2022; Seidl et al. 2015; Smith et al. 2017).

The genomes of several obligate biotrophic plant‐pathogenic lineages, including powdery mildew fungi, rust fungi, and some oomycetes, are characterised by the loss of primary metabolic pathways such as thiamine biosynthesis and the inability to assimilate inorganic sulfur and nitrogen (Baxter et al. 2010; Duplessis et al. 2011; Spanu 2012; Spanu et al. 2010; Sabelleck, Freh, et al. 2025). Since the *Arachnopezizaceae* are closely related to powdery mildew fungi, we queried *A. aurata* and *A. aurelia* for components of primary metabolism. Their primary metabolism profiles were more similar to that of the saprobic and necrotrophic fungus *B. cinerea* , including the presence of the thiamine biosynthesis pathway and sulfur and nitrogen assimilation pathways (Figure 4A). This is consistent with the ability of *A. aurata* and *A. aurelia* to grow under in vitro conditions (Figure 1). Further, obligate biotrophic plant pathogens like the powdery mildew fungi harbour genomes inflated in size due to the massive expansion of TEs, associated with the loss of the RIP pathway to control TE spread (Kusch, Qian, et al. 2024; Spanu et al. 2010). Both *A. aurata* and *A. aurelia* have average‐sized genomes exhibiting TE contents below 5% (Table 1) and evidence of a functional RIP mechanism (Figure 3). TEs are preferentially located in gene‐sparse regions with low GC content (Figure 2), consistent with a general AT‐richness of TE‐derived open reading frames (Jia and Xue 2009) and observations that TEs often cluster in gene‐poor genomic regions in, for instance, the plant pathogen *Zymoseptoria tritici* (Oggenfuss and Croll 2023). Hence, the genome architectures of *A. aurata* and *A. aurelia* do not resemble those of obligate biotrophic plant pathogens like the powdery mildew fungi.

Saprobic fungi rely on a combination of secreted plant cell wall‐degrading enzymes and the non‐enzymatic Fenton reaction to decompose lignin and complex polysaccharides in wood and plant litter (Eastwood et al. 2011). Saprotrophic as well as necrotrophic fungi employ a wide array of CAZymes to degrade the complex polysaccharides derived from plant cell walls (Hage and Rosso 2021), although the number of CAZymes can vary widely in ascomycetes, irrespective of their lifestyle (Zhao et al. 2013). In line with a saprotrophic lifestyle and in contrast to the obligate biotrophic barley powdery mildew pathogen *B. hordei*, both *A. aurata* and *A. aurelia* have more than 500 proteins predicted to function as CAZymes (Figure 4B). Different from *B. hordei*, the CAZyme repertoire includes numerous glycosyl hydrolases and accessory enzymes (Figure 4B). This CAZyme profile resembles other saprotrophic fungi such as *Verticillium tricorpus* (Seidl et al. 2015). Taken together, *A. aurata* and *A. aurelia* exhibit the characteristic genomic signatures of a saprotrophic fungus able to decompose plant cell walls, and their genomes do not resemble the transposon‐enriched architecture of the obligate biotrophic powdery mildew fungi.

Contrasting the genomic features expected of a saprotrophic fungus, we found more than 400 predicted effectors in each of the proteomes of *A. aurata* and *A. aurelia* (Figure 4C; Table 2). While functions related to carbohydrate modification and proteases were predicted for some of these candidate effectors (Tables S2 and S3), which is consistent with a saprobic lifestyle involving the decomposition of organic plant matter, around half of the proteins in both *Arachnopeziza* species had no predicted function and/or recognisable protein domain. However, the effector and CAZyme content of the genome alone may not always suffice to distinguish fungi of saprotrophic and pathogenic lifestyles. For instance, saprotrophic and plant‐pathogenic *Fusarium* species exhibit a large overlap in effector and CAZyme repertoires, and lineages can be rather distinguished by a few lineage‐specific effectors (Hill et al. 2022). In another example, the genome of the saprotrophic fungus *V. tricorpus* contains an effector repertoire similar to that of the vascular wilt pathogen *V. dahliae*, but an expanded set of CAZymes (Seidl et al. 2015). *V. tricorpus* is a saprotrophic fungus, but occasionally transitions to a pathogenic lifestyle (Farag et al. 2024; Nair et al. 2015). While such opportunistic infections by *Arachnopeziza* are not described, the large number of effectors in *A. aurata* and *A. aurelia* could facilitate pathogenic or mutualistic interactions with plants under certain conditions.

Several *Arachnopeziza* species seem to be closely associated with mosses such as *Sphagnum* and liverworts like *Ptilidium* (Kosonen et al. 2020; Stenroos et al. 2010). It is currently unclear if these associations represent close mutualistic or pathogenic interactions between *Arachnopeziza* species and mosses or liverworts. One possibility is that some of the potential effectors in *A. aurata* and *A. aurelia* serve to facilitate the interaction or communication with mosses. However, the species do not exhibit any of the auxotrophies that are typical for obligate biotrophic plant pathogens such as powdery mildews and rusts (Figure 4A). Likewise, *A. aurata* and *A. aurelia* do not seem to possess any polysaccharide lyases, which cleave uronic acid‐containing polysaccharide chains (Lombard et al. 2010) and belong to a family of CAZymes often expanded in plant pathogens but absent in saprobic fungi (Karlsson et al. 2015). Therefore, such interactions may be either facultative or commensal. Indeed, saprotrophic fungi may harbour the capacity to enter into facultative biotrophic relationships with plant roots without causing disease symptoms (Smith et al. 2017), for instance by regulating their plant cell wall‐degrading enzymes (Olson et al. 2012), which may otherwise cause activation of the plant immune system (Plett and Martin 2011). A detailed study of the association between mosses and liverworts with *Arachnopeziza* is required to shed light on the quality of the interaction and has the potential to help better understand the saprobic and biotrophic lifestyles of these fungi.

Effectors can also mediate interactions with other microbes. For instance, the *V. dahliae* effectors VdAve1 and VdAMP2 modulate the microbiota composition within and outside the host plant, possibly to suppress harmful competitors or toxin‐producing microbes (Snelders et al. 2020). Likewise, *A. aurelia* and *A. aurata* may employ effectors to help them compete or cooperate with other fungi and bacteria (Snelders et al. 2022).

Functional studies of powdery mildew effectors and other proteins are currently limited to indirect methods such as host‐induced gene silencing (Nowara et al. 2010; Pedersen et al. 2012) and the screening of EMS‐ or UV‐mutagenised populations (Barsoum et al. 2020; Bernasconi et al. 2024). Some attempts to genetically modify powdery mildew fungi in a targeted manner have been reported (Chaure et al. 2000; Martínez‐Cruz et al. 2017), but a reliable and reproducible transformation system is lacking to date, possibly due to the obligate biotrophic lifestyle preventing in vitro cultivation. Here, we report that *A. aurata* , a saprotrophic fungus belonging to the family of the closest living relatives of powdery mildew fungi, can be genetically modified using a well‐established protoplast‐based transformation protocol (Leisen et al. 2020; Figure 6). We argue that *A. aurata* holds great promise to study at least some of the powdery mildew proteins functionally, for instance concerning subcellular localisation using fluorescent tags, protein–protein interactions, or post‐translational modification profiles, all of which are currently not possible to assess in the fungus and can be only indirectly analysed by *in planta* expression (e.g., Pennington et al. 2019). Further, targeted auxotrophies of, for instance, thiamine or the inability to assimilate sulfur or nitrogen could be introduced to study the genetic basis of obligate biotrophy in powdery mildew fungi.

## Author Contributions

R.P., L.K., and S.K. designed the study; S.K. was responsible for experiment conception and planning. S.K. established the fungal in vitro cultures, supervised RNA and DNA extractions, and supervised cloning and genetic modification attempts. A.L. performed high molecular weight genomic DNA extraction and long‐read DNA sequencing, established genetic modification protocols, and cloning and genotyping of strains. E.D. reproduced the genetic modification protocol, cloning, and genotyping. F.K. generated the samples for RNA‐sequencing used for gene annotation. J.Q. conducted repeat masking and TE analysis of the *Arachnopeziza* genomes. H.I. did KEGG analysis. S.K. performed genome assemblies and polishing, annotation, comparative genomics, and data analysis. S.K. drafted figures and wrote the first draft of the manuscript, and S.K., L.K., and R.P. edited the manuscript. All authors read the manuscript and approved the final version.

## Conflicts of Interest

The authors declare no conflicts of interest.

## Supporting information


**Figure S1:** The mitochondrial genomes of *A. aurata* and *A. aurelia* are compact and seem to lack the ATP synthase component *atp8*. The circular mitochondrial genome assemblies were recovered from the initial whole genome assemblies of *A. aurata* CBS127647 (left) and *A. aurelia* CBS127675 (right). The mitochondrial genes were annotated using MFannot (Lang et al. 2023) and additional BLASTN and TBLASTN searches to recover missing components. The thick bars indicate the coding genes for the mitochondrial respiratory machinery colour‐coded by the mitochondrial respiratory complex, that is, NADH:ubiquinone oxidoreductase (complex I, orange), ubiquinol‐cytochrome c oxidoreductase (complex III or *bc*_1_ complex, dark orange), cytochrome c oxidase (complex IV, yellow), ATP synthase (green), and ribosomal protein *rps3* (dark blue); other open reading frames are shown in grey. Thin bars indicate noncoding RNAs (*rns*, small ribosomal RNA, light blue; *rnl*, large ribosomal RNA, light blue; *rnpB*, mitochondrial RNaseP‐RNA, purple). The mitochondrial tRNAs are added as labels.
**Figure S2:** The genomes of *Arachnopeziza aurata* and *A. aurelia* show signs of TE degeneration via RIP. We performed RIP index analysis based on dinucleotide ratios via RIPCAL (Hane and Oliver 2008) on the genome assemblies of *A. aurata* CBS127674 and *A. aurelia* CBS127675 with the assemblies of the barley powdery mildew *Blumeria hordei* DH14 (Frantzeskakis et al. 2018) and the barley leaf scald disease pathogen *Rhynchosporium commune* UK7 (Penselin et al. 2016). Panels are displayed in this order from left to right. The bar graphs show the ratios of repetitive sequence dinucleotide analysis genome‐wide (dark grey), for coding sequences (CDS; light grey), *Ty1*/*Copia* LTR elements (yellow), *Ty3*/*mdg4* LTR elements (orange), and DNA transposons (blue). The respective RIP index represented by dinucleotide ratios is indicated on the x‐axis, and the y‐axis displays the respective ratio.
**Figure S3:** Proteins of *Arachnopeziza aurata* and *A. aurelia* with ribonuclease‐like domains are distinct from RNase‐like powdery mildew effectors. *A. aurata* and *A. aurelia* putative secreted proteins containing PFAM domains (PF00445, PF06479) or InterPro domains (IPR036430, IPR016191) from the Ribonuclease T2‐like superfamily were queried against the BLAST non‐redundant protein database (nr on https://blast.ncbi.nlm.nih.gov/Blast.cgi accessed 04/2025) and protein sequences with similarity extracted for alignment. In addition, three RNase‐like effector proteins (AVRPM2 of *B. graminis* f.sp. *tritici* and CSEP0064 and CSEP0264 of *B. hordei* (Spanu 2017)) were included for multiple sequence alignment. Alignment and phylogenetic reconstructions were performed using the function ‘build’ of ETE3 3.1.3 (Huerta‐Cepas et al. 2016) as implemented on the GenomeNet (https://www.genome.jp/tools/ete/; accessed 04/2025). The maximum likelihood (ML) tree was inferred using RAxML v8.2.11 with model PROTGAMMAJTT and default parameters (Stamatakis 2014). Branch supports were calculated using a Shimodaira‐Hasegawa‐like approximate likelihood ratio test, a method for assessing the statistical support of different tree topologies (SH‐like values). The NCBI GenBank accessions and respective species are indicated; AAUREL indicates *A. aurelia* and AAURAT *A. aurata* proteins. IRE1, Inositol‐requiring protein 1 (IRE1); RALPH, RNase‐like proteins associated with haustoria; RNase, ribonuclease.
**Figure S4:** The genomes of *Arachnopeziza aurata* and *A. aurelia* harbour complements of secondary metabolite biosynthesis gene clusters throughout their genomes. We detected components of secondary metabolism using antiSMASH 7.0 (Blin et al. 2023; Medema et al. 2011) and mapped the respective locations of the coding genes on the genomic maps of *A. aurata* (A) and *A. aurelia* (B), displayed as circos plots generated with the R package circlize v0.4.10 (Gu et al. 2014). The outer tracks indicate the chromosomal map and the scale the chromosomal position in million base pairs (Mbp). The labels denote the locations of components of secondary metabolism, which were Type 1 polyketide synthase (T1PKS), non‐ribosomal peptide synthetase (NRPS, NRPS‐like), fungal ribosomally synthesised and post‐translationally modified peptide (fungal‐RiPP‐like), terpene biosynthesis (Terpene), and indole biosynthesis (Indole).
**Figure S5:** Alignment of AAUREL_003350‐RA with different ribonucleases of the T1 family and ribonuclease‐like effectors of plant‐pathogenic fungi. The multiple sequence alignment of the amino acid sequences was generated with SnapGene v6.0.2 and the implemented MAFFT v7.471 aligner (SnapGene software (from Dotmatics; available at snapgene.com)). The residues required for guanyl‐specific ribonuclease activity of full‐length RNase T1 are H66, E84, R103, H118, which correspond to P40, E58, R77, H92, respectively (numbering without signal peptide). Sequences and Uniprot identifiers: RNase T1 (P00651), RNase F1 (P10282), RNase ms (P00653), RNase alpha‐sarcin (P00655), Hirsutellin A (P78696), ZT6 (F9X693), SRE1 (R0K2C5), FG12 (I1S329), AVRA1 (N1JGD1), AVRA6 (A0A383UZH9), AVRA7 (N1J8Q9), AVRA9 (N1J9M0), AVRA10 (N1J9C5), AVRA13 (N1JFM8), AVRA22 (N1J9C5), AVRPm2 (A0A1L5JEG4). Amino acid sequence conservation compared to RNase T1 is highlighted in yellow; red Arrows indicate catalytic residues required for ribonuclease activity.
**Figure S6:** *Arachnopeziza aurata* and *A. aurelia* are inhibited by common fungicides. The strains *Arachnopeziza aurata* CBS127674 (left) and *A. aurelia* CBS127675 (right) were cultivated on potato dextrose agar (PDA) containing (A) fenhexamid, (B) kanamycin, (C) ampicillin, (D) cefotaxime, (E) streptomycin, or (F) neomycin at the indicated concentrations. The plates were incubated at 23°C; photographs were taken 16 days after inoculation. Note that the control pictures for the 0 μg mL^−1^ negative control are identical if the respective fungicides/antibiotics were tested in the same experiment.
**Figure S7:** Genotyping PCRs for transgenes in *Arachnopeziza aurata*.
*A. aurata* CBS127674 was genetically modified using a modified PEG‐mediated protoplast transformation protocol (Leisen et al. 2020; Wegner et al. 2022). Transgenic strains are denoted by an H (pTK144; hygromycin resistance) or F (pTelFen, fenhexamid resistance). Genotyping PCRs for (A) lines H1‐H3, (B) Lines H4‐H6 and F1‐F7, and (C) lines H1 and F1 are displayed for the transgenes *HPH*, hygromycin resistance gene; *mRFP*, red fluorescent protein gene; *FfERG27*, fenhexamid resistance allele of *Fusarium fujikuroi*. Two endogenous *A. aurata* genes were genotyped as control, that is, *TUB2*, *A. aurata* tubulin 2‐coding gene; *GAPDH*, A. aurata glyceraldehyde 3‐phosphate dehydrogenase‐coding gene. Oligonucleotides are listed in Expected amplicon sizes were: *HPH*, 692 bp; *mRFP*, 591 bp; *FfERG27*, 648 bp; *TUB2*, 654 bp; *GAPDH*, 632 bp. P, positive control plasmids pTK144 (A and C, upper panel) and pTelFen (lower panel); H2O, no template negative control; NPC, no primer control. Size marker, 1 kb plus (Invitrogen‐Thermo Fisher, Waltham, MA, USA).

**File S1:** BLASTN search results for 5.8S, 18S, 28S nuclear ribosomal DNA (nrDNA) and ITS sequences (accessions MH864617.1, MH876055.1, MH864618.1, and MH876056.1) in the genome of *Arachnopeziza aurata*.

**File S2:** BLASTN search results for 5.8S, 18S, 28S nuclear ribosomal DNA (nrDNA) and ITS sequences (accessions MH864617.1, MH876055.1, MH864618.1, and MH876056.1) in the genome of *Arachnopeziza aurelia*.

**File S3:** TBLASTN search results for the repeat‐induced point mutation (RIP) proteins *Masc1*, *Masc2*, *Rid‐1*, and *Dim‐2* (GenBank accession numbers AAC49849.1, AAC03766.1, XP_011392925.1, and XP_959891.1, respectively) in the genome of *Arachnopeziza aurata*.

**File S4:** TBLASTN search results for the repeat‐induced point mutation (RIP) proteins *Masc1*, *Masc2*, *Rid‐1*, and *Dim‐2* (GenBank accession numbers AAC49849.1, AAC03766.1, XP_011392925.1, and XP_959891.1, respectively) in the genome of *Arachnopeziza aurelia*.

**File S5:** Multiple sequence alignment of *Arachnopeziza aurelia* AAUREL_003350‐RA with different ribonucleases of the T1 family and ribonuclease‐like effectors of plant pathogenic fungi.

**File S6:** Multiple sequence alignment of protein sequences from *Arachnopeziza aurata* and *A. aurelia* containing ribonuclease‐like domains, RNase‐like effectors of *Blumeria hordei* and *B. graminis* f.sp. *tritici*, and ribonuclease‐like proteins identified by a BLASTP search at https://blast.ncbi.nlm.nih.gov/Blast.cgi (accessed 04/2025).

**Table S1:** Oligonucleotides used in this study.
**Table S2:** Functional predictions of *Arachnopeziza aurata* proteins annotated by BRAKER3.
**Table S3:** Functional predictions of *Arachnopeziza aurelia* proteins annotated by BRAKER3.
**Table S4:** KEGG pathway mapping summary of *Arachnopeziza aurata* and *A. aurelia* compared to *Botrytis cinerea* B05.10 and *Blumeria hordei* DH14.
**Table S5:** Orthogroups of putative secreted proteins of *Arachnopeziza aurata*, *A. aurelia* , and *Blumeria hordei* DH14.
**Table S6:** List of *Arachnopeziza* proteins containing ribonuclease‐like domains.

## Data Availability

All raw RNA and DNA sequencing data generated in this study are deposited at https://www.ncbi.nlm.nih.gov/sra under BioProject ID PRJNA1128938. The draft genome assemblies for *Arachnopeziza aurata* CBS127674 and *A. aurelia* CBS127675 have been deposited at DDBJ/ENA/GenBank (accessions GCA_051247085.1 and GCA_051247835.1, respectively). Genome assemblies and gene annotations as used in this work are available at https://doi.org/10.5281/zenodo.15303401.
